# Frozen-Density Embedding for Including Environmental Effects in the Dirac-Kohn–Sham Theory: An Implementation Based on Density Fitting and Prototyping Techniques

**DOI:** 10.1021/acs.jctc.2c00499

**Published:** 2022-09-29

**Authors:** Matteo De Santis, Diego Sorbelli, Valérie Vallet, André Severo Pereira Gomes, Loriano Storchi, Leonardo Belpassi

**Affiliations:** † Univ. Lille, CNRS, UMR 8523-PhLAM-Physique des Lasers Atomes et Molécules, F-59000 Lille, France; ‡ Dipartimento di Chimica, Biologia e Biotecnologie, Università degli Studi di Perugia, Via Elce di Sotto 8, 06123 Perugia, Italy; ¶ Istituto di Scienze e Tecnologie Chimiche (SCITEC), Consiglio Nazionale delle Ricerche c/o Dipartimento di Chimica, Biologia e Biotecnologie, Università degli Studi di Perugia, Via Elce di Sotto 8, 06123 Perugia, Italy; § Dipartimento di Farmacia, Università degli Studi ‘G. D’Annunzio’, Via dei Vestini 31, 66100 Chieti, Italy

## Abstract

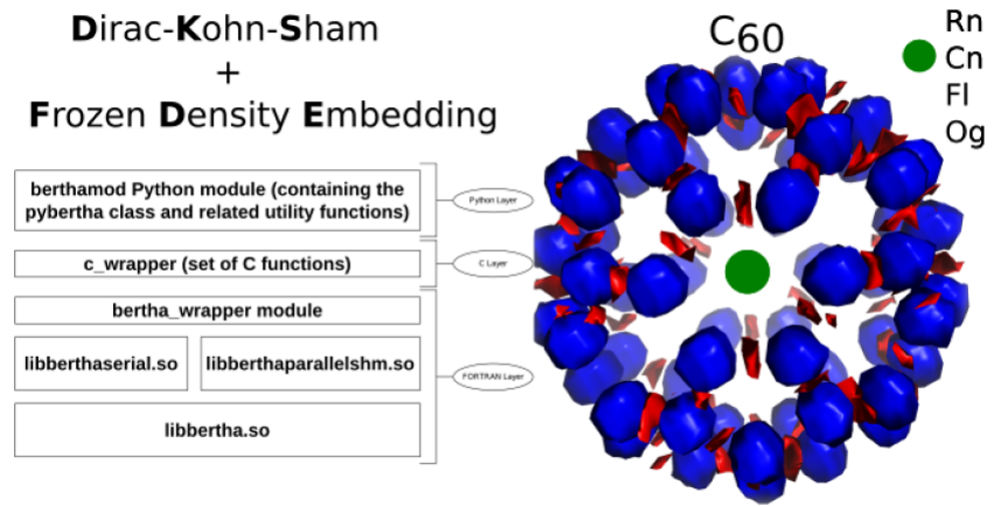

Frozen density embedding (FDE) represents an embedding scheme in which environmental effects are included from first-principles calculations by considering the surrounding system explicitly by means of its electron density. In the present paper, we extend the full four-component relativistic Dirac–Kohn–Sham (DKS) method, as implemented in the BERTHA code, to include environmental and confinement effects with the FDE scheme (DKS-in-DFT FDE). The implementation, based on the auxiliary density fitting techniques, has been enormously facilitated by BERTHA’s python API (PyBERTHA), which facilitates the interoperability with other FDE implementations available through the PyADF framework. The accuracy and numerical stability of this new implementation, also using different auxiliary fitting basis sets, has been demonstrated on the simple NH_3_–H_2_O system, in comparison with a reference nonrelativistic implementation. The computational performance has been evaluated on a series of gold clusters (Au_*n*_, with *n* = 2, 4, 8) embedded into an increasing number of water molecules (5, 10, 20, 40, and 80 water molecules). We found that the procedure scales approximately linearly both with the size of the frozen surrounding environment (consistent with the underpinnings of the FDE approach) and with the size of the active system (in line with the use of density fitting). Finally, we applied the code to a series of heavy (Rn) and super-heavy elements (Cn, Fl, Og) embedded in a C_60_ cage to explore the confinement effect induced by C_60_ on their electronic structure. We compare the results from our simulations, with respect to more-approximate models employed in the atomic physics literature. Our results indicate that the specific interactions described by FDE are able to improve upon the cruder approximations currently employed, and, thus, they provide a basis from which to generate more-realistic radial potentials for confined atoms.

## 1 Introduction

Molecular systems, clusters, and materials containing heavy atoms have drawn considerable attention recently, because of their rich chemistry and physics.^1−4^ In order to model computationally systems containing heavy elements, the methods of relativistic quantum mechanics must be necessarily adopted to capture scalar and spin–orbit interactions that are neglected in the conventional nonrelativistic formulation of quantum chemistry. Furthermore, most of the chemistry occurs in solution and the environment plays a key role in the determination of the properties and reactivity of substances in condensed phases.^5−9^ Thus, the complexity of chemical phenomena in solution has made it necessary to develop a variety of models and computational techniques to be combined with (relativistic) quantum chemistry methods. Among the different approaches to include environmental effects, we mention the quantum mechanics/molecular mechanics (QM/MM) approach,^10^ which includes the molecular environment explicitly and at a reduced cost using a classical mechanical description, or in a polarizable continuous medium (PCM)^11^ (i.e., where the solvent degrees of freedom are replaced by an effective classical dielectric). Despite being widely and successfully applied, these methods may have drawbacks. For instance, methods based on PCM cannot describe specific interactions with the environments (e.g., hydrogen, halogen bonds), while the QM/MM approach, which is based on classical force fields, may be limited by the availability of accurate parametrizations which may reduce its predictive power, in particular when heavy elements are involved. An alternative is to use quantum embedding theories (for an overview, see refs (12−15) and references therein), in which a QM description for a subsystem of interest is combined with a QM description of the environment (QM/QM). A notable example of QM/QM methods is the frozen-density embedding (FDE) scheme introduced by Wesołowski and Warshel,^16,17^ based on the approach originally proposed by Senatore and Subbaswamy,^18^ and later Cortona,^19^ for solid-state calculations. The method has been further generalized^20,21^ and directed to the simultaneous optimization of the subsystems electronic densities.

FDE is a DFT-in-DFT embedding method that allows one to partition a larger Kohn–Sham system into a set of smaller, and coupled, Kohn–Sham subsystems. The coupling term is defined by a local embedding potential, depending only on the electron densities of both the sole active subsystem and the environment (i.e., no orbital information is shared among subsystems). This feature gives to the FDE scheme an enormous flexibility, as indeed virtually arbitrary methods can be combined to treat different subsystems. For example, wave function theory (WFT)-based methods can be used for the active system while one can take advantage of the efficiency of DFT to describe a large environment (WFT-in-DFT).^12,13,17,22−25^ Also one can employ very different computational protocols for different subsystems including (i) using Hamiltonian dealing with different relativistic approximations (from the full four-component methods to the nonrelativistic ones);^26−29^ (ii) different basis sets size and type (Gaussian- and Slater-type functions, relativistic four-component spinors), and even (iii) different quantum chemical packages.^26,30,31^ We mention that the FDE scheme has been extended both to the linear-response TDDFT,^32−34^ including to account for charge-transfer excitations^35,36^ and to real-time TDDFT (rt-TDDFT).^31,37^

FDE-based calculations are shown to be accurate in the case of weakly interacting systems including hydrogen bond systems,^38,39^ whereas their use for subsystems interacting with a larger covalent character is problematic (see ref (38) and references therein), because of the use of approximate kinetic energy functional (KEDF) in the nonadditive contribution to the embedding potential. The research for more accurate KEDFs is a key aspect for the applicability of the FDE scheme as a general scheme,^40−43^ including the partitioning of the system also breaking covalent bonds.^44^ We mention here that alternative QM/QM approaches, avoiding the use of KEDFs and also allowing for fragmentation in subsystems through covalent bonds, have been recently proposed (see, for instance, refs (14,15,45−51)).

Thanks to its flexibility, the FDE scheme has been implemented in different flavors into computational packages such as embedded Quantum Espresso,^52^ ADF,^21,53,54^ Turbomole,^55,56^ Dalton,^26,57^ Koala,^58^ Molpro,^45^ Serenity,^59^ and Q-Chem,^60^ (the first two are based on plane waves and Slater-type functions, respectively; the others are based on Gaussian-type functions). FDE has also been implemented to treat the subsystems at full relativistic four-component level based on the Dirac equation within the DIRAC code,^61^ and can be used with DFT and different wave function methods both for molecular properties and energies involving the ground or excited electronic states.^26,28,29,62−64^

Despite its conceptual simplicity, its actual implementations may lead to relatively complicated workflows. Therefore, a simpler approach is to integrate such legacy codes as computational engines to handle the different FDE steps, which are then glued together and their execution automatized using suitable frameworks, such as that implemented in PyADF,^30,65^ that can be easily extensible, because of its object-oriented implementation in the Python programming language.^66^ Prototyping techniques also based on Python are very useful to build reference implementations, for instance, the Psi4-rt-PyEmbed code,^31,67^ where the Python interface of Psi4Numpy and PyADF^30,68^ (including its PyEmbed module^69,70^ and XCFun library^71,72^ to evaluate nonadditive exchange-correlation and kinetic energy contributions) has been used by some of us to build real-time nonrelativistic TDDFT-in-DFT FDE^31^ and projection-based embedding^73^ implementations.

In this work, we extend the Dirac–Kohn–Sham (DKS) method implemented in the BERTHA code (with its new Python API, PyBERTHA)^74,75^ to the FDE scheme to include environmental/confinement effects in the DKS calculations (DKS-in-DFT FDE). The implementation takes advantage of the DKS formulation implemented in BERTHA, including the density fitting algorithms at the core of the computation (i.e., in the evaluation of the embedding potential and of its matrix representation is relativistic G-spinor functions), and the FDE implementation already available in the PyEmbed module of the PyADF framework. The auxiliary density fitting scheme presented here reduces the scaling of the numerical integration step of the evaluation of the embedding potential matrix representation, avoiding the numerical integration over principal spinor basis set amplitudes, which is typically employed in other four-component relativistic implementations.^26^

The outline of the paper is as follows. In section 2, we present the basic theory of FDE and a brief description of the DKS method as implemented in BERTHA. In section 3, we then describe, in detail, our implementation. In section 4, we present some numerical results, including the computational burden and scalability of this new implementation, with respect to the size of the active system, as well as of the embedding one. We will also present an application to a series of heavy elements (Rn) and super-heavy elements (Cn, Fl, Og) confined into a C_60_ cage. Finally, concluding remarks are given in section 5.

## 2 Theory

In this section, we briefly review the basic formalism of the FDE scheme and its extension to use the DKS theory for the active system (DKS-in-DFT FDE). We will also address some details of the DKS implementation in BERTHA, mainly focusing on those aspects (including density fitting techniques), which are relevant for an efficient implementation of the FDE scheme. Finally, we will illustrate the basic characteristics of the recent BERTHA Python API, PyBERTHA (and the related **pyberthamod** module available under GPLv3 license in ref (67); for additional and technical details, see refs (74−76)), which is a key tool here to devise a simple workflow for the DKS-in-DFT FDE scheme.

### 2.1 Subsystem DFT and Frozen Density Embedding Formulation

In the subsystem formulation of DFT, the entire system is partitioned into *N* subsystems, and the total density (ρ_tot_(***r***)) is represented as the sum of electron densities of the various subsystems [i.e., ρ_*a*_(***r***) (*a* = *I*, .., *N*)]. In the following, we consider the total density as partitioned in only two contributions as
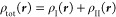
1The total energy of the system can then be written as
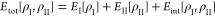
2with the energy of each subsystem (*E*_*i*_[ρ_*i*_], with *i* = I, II), given according to the usual definition in DFT as
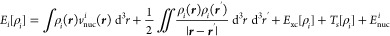
3In the above expression, *v*_nuc_^*i*^(***r***) is the nuclear potential due to the set of atoms that defines the subsystem, and *E*_nuc_^*i*^ is the related nuclear repulsion energy. *T*_s_[ρ_*i*_] is the kinetic energy of the auxiliary noninteracting system, which is, within the Kohn–Sham (KS) approach, commonly evaluated using the KS orbitals. The interaction energy is given by the expression
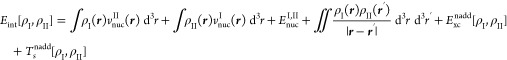
4with *v*_nuc_^I^ and *v*_nuc_^II^ being the nuclear potentials due to the set of atoms associated with subsystems I and II, respectively. The repulsion energy for nuclei belonging to different subsystems is described by the *E*_nuc_^I,II^ term. The nonadditive contributions (*E*_xc_^nadd^[ρ_I_, ρ_II_] and *T*_*s*_^nadd^[ρ_I_, ρ_II_]) arise because both exchange-correlation and kinetic energy, in contrast to the Coulomb interaction, are not linear functionals of the density.

The electron density of a given fragment (ρ_I_ or ρ_II_ in this case) can be determined by minimizing the total energy functional (eq 2), with respect to the density of the fragment while keeping the density of the other subsystem frozen. This procedure is the essence of the FDE scheme and leads to a set of Kohn–Sham-like equations (one for each subsystem)
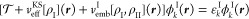
5which are coupled by the embedding potential term *v*_emb_^I^(***r***). The latter carries all dependence on the other fragment’s density. Here,  denotes the kinetic energy operator, which, in a nonrelativistic framework, has the form −∇^2^/2, whereas, for a relativistic framework, it is *c***α** · **p** (see discussion below). We also note that in the relativistic framework, the FDE expressions above correspond to the case in which an external vector potential is absent. Further details for their generalization can be found in ref (28).

In this equation, *v*_eff_^KS^[ρ_I_](***r***) is the KS potential calculated on the basis of the density of subsystem I only, whereas *v*_emb_^I^[ρ_I_, ρ_II_](***r***) is the embedding potential that takes into account the effect of the other subsystem (which we consider here as the complete environment). In the framework of FDE theory, *v*_emb_^I^[ρ_I_, ρ_II_](***r***) is explicitly given by
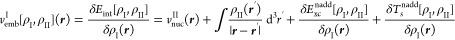
6where the nonadditive exchange-correlation and kinetic energy contributions are defined as the difference between the associated exchange correlation and the kinetic potentials defined using ρ_tot_(***r***) (i.e., ρ_I_(***r***) + ρ_II_(***r***)) and ρ_I_(***r***). For both potentials, one must account for the fact that only the density is known for the total system, so that potentials that require input in the form of KS orbitals are prohibited. For the exchange-correlation potential, one may make use of accurate density functional approximations and its quality is therefore similar to that of ordinary KS. The potential for the nonadditive kinetic term (, in eq 6) is more problematic as less accurate orbital-free kinetic energy density functionals (KEDFs) are available for this purpose. Examples of popular functional approximations applied in this context are the Thomas–Fermi (TF) kinetic energy functional^77^ or the GGA functional PW91k.^78^ As already mentioned in the Introduction, the research for more accurate KEDFs is a key aspect for the applicability of the FDE scheme as a general scheme, including the partitioning of the system also breaking covalent bonds.^44^

Generally, the set of coupled equations that arises in the FDE scheme for the subsystems must be solved iteratively with a freeze-and-thaw scheme, where one relaxes the electron density of one subsystem at a time keeping frozen the others, until electron densities of all subsystems reach a required convergence. The implementation of the freeze-and-thaw method employing the actual DKS electron density requires a switching between the active/frozen subsystems and, in this specific case, an efficient evaluation of the Coulomb potential from the DKS calculation on a numerical grid, which is currently under development in our laboratory.

In this work, we will limit ourselves to one subsystem (active) while keeping the density of the environment frozen to their ground-state density. The scheme is suitable for neutral weakly interacting subsystems, although it neglects, by definition, possible mutual polarization, which it is known to be important in those cases in which one or more subsystems possess a net charge or important higher electric multipoles. Considering only one active subsystem, the implementation of FDE reduces to the evaluation of *v*_emb_^I^(***r***) potential (which is a one-electron operator) that must be added to the Hamiltonian of the active system. The matrix representation of the embedding potential may be evaluated using numerical integration grids, similar to those used for the exchange-correlation term in the KS method. This contribution is then added to the KS matrix and the eigenvalue problem is solved with the usual self-consistent field (SCF) procedure. We note here that two approaches can be taken: the first is the use of a precalculated embedding potential^26^ (for instance, from a prior subsystem DFT calculation) that is used as a one-body operator (referred to in the literature as a “static” embedding potential) added to the one-body Fock matrix at the start of the (four-component) calculations. The second approach involves the regeneration of the embedding potential using the (four-component) actual electron density of the active system. In this case, the matrix representation of the embedding potential is updated during SCF procedure, because of its dependence on the active subsystem density (see eq 6) that itself changes during the SCF iterations. As discussed below, in this work we shall mostly make use of the latter approach.

### 2.2 Dirac–Kohn–Sham Scheme in BERTHA and Its Extension to FDE Based on Density Fitting

For the detailed theoretical basis of the Dirac–Kohn–Sham methodology, we refer the reader to previous works^79−85^ and references therein. Here, we summarize only the main aspects of the DKS method based on the use of G-spinor basis sets and the density-fitting techniques as implemented in BERTHA.^75,86^ In atomic units, and including only the longitudinal electrostatic potential, the DKS equation reads
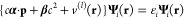
7where *c* is the speed of light in a vacuum and **p** is the electron momentum, while
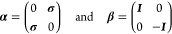
8where **σ** = (σ_*x*_, σ_*y*_, σ_*z*_), σ_*q*_ is a 2 × 2 Pauli spin matrix and ***I*** is the 2 × 2 identity matrix. The longitudinal interaction term is represented by a diagonal operator borrowed from nonrelativistic theory and is composed of a nuclear potential term *v*_N_(***r***), a Coulomb interaction term *v*_H_^(*l*)^[ρ(***r***)], and the exchange-correlation term *v*_xc_^(*l*)^[ρ(***r***)]. We mention that the Breit interaction contributes to the transverse part of the Hartree interaction and is not considered here, as we restrict ourselves to using nonhybrid, nonrelativistic functionals of the electron density.

In BERTHA, the spinor solution (**Ψ**_*i*_(**r**) in eq 7) is expressed as a linear combination of the G-spinor basis functions,^87^*M*_μ_^*T*^(***r***) (*T* = L, S where L and S refer to the so-called “large” and “small” components, respectively). The G-spinors do not suffer from the variational problems of kinetic balance (see ref (88) and references therein) and, regarding the evaluation of multicenter integrals, retain the advantages that have made Gaussian-type functions the most widely used expansion set in nonrelativistic quantum chemistry. The matrix representation of the DKS operator in the G-spinor basis is given by
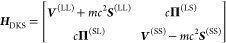
9where
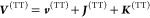
10The eigenvalue equation in the algebraic representation is given by
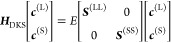
11where ***c***^(T)^ are the spinor expansion vectors. The ***H***_DKS_ matrix is defined in terms of the ***v***^(TT)^, ***J***^(TT)^, ***K***^(TT)^, ***S***^(TT)^, and **Π**^(TT′)^ matrices, being, respectively, the basis representation of the nuclear, Coulomb, and exchange-correlation potentials, the overlap matrix, and the matrix of the kinetic operator, respectively. The nuclear charges have been modeled by a finite Gaussian distribution.^89^

The resulting matrix elements are defined by
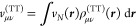
12
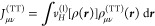
13
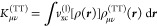
14
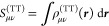
15
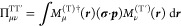
16The terms ρ_μ*ν*_^(TT)^(**r**) are the G-spinor overlap densities (*M*_μ_^(T)†^(**r**)*M*_ν_^(T)^(**r**)), which can be exactly expressed as linear combination of standard Hermite Gaussian-type functions (HGTFs).^86,87,90^ The ***H***_DKS_ matrix is dependent on ρ(***r***) in *v*_xc_^(l)^[ρ(***r***)] and *v*_H_^(l)^[ρ(***r***)], through the canonical spinors obtained by its diagonalization. Thus, the solutions ***c***^(T)^ are solved self-consistently.

In the G-spinor representation, we define the density matrix (***D***^(TT′)^) as the product column by row of the *c*_μ_^(T)^ coefficients (*D*_μ*ν*_^(TT′)^ = ∑_*i*_*c*_μ*i*_^(T)*^c_*νi*_^(T′)^, with T and T′ equal to both L and S), where the sum runs over the occupied positive-energy states. The total electron density is obtained according to the expression
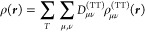
17The computation of the Coulomb and exchange-correlation contributions to the DKS matrix, that is, eqs 13 and 14, respectively, is the most demanding computational step in a DKS calculation involving a G-spinor basis set. The current version of BERTHA takes advantage of both density fitting^86,91−93^ and advanced parallelization techniques^74,75,94−96^ for the evaluation of these two contributions. The relativistic density (which is a real scalar function) is thereby expanded in a set of *N*_aux_ auxiliary atom-centered functions.
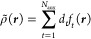
18

In the Coulomb metric, the expansion coefficients *d*_*t*_ are defined as the solution of the linear system, given by
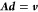
19where ***A*** is a real and symmetric matrix, representing the Coulomb interaction in the auxiliary basis, *A*_*st*_ = ⟨*f*_s_| |*f*_t_⟩ while the elements (*v*_s_) of the vector ***v*** are the projection of the electrostatic potential on the fitting functions,
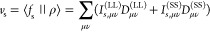
20which can be expressed in terms of the density matrix elements *D*_μ*ν*_^(TT)^ and of the three-center two-electron repulsion integrals
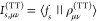
21involving the auxiliary fitting functions and the G-spinor overlap densities, ρ_μ*ν*_^(TT)^(**r**). In our implementation, the calculation of elements, *v*_s_, can be also be efficiently evaluated using the relativistic generalization^91^ of the scalar Hermite density matrix proposed by Almlöf.^97,98^ The evaluation of the Coulomb matrix is given by
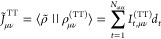
22The procedure involves only the evaluation of two-center Coulomb integrals over the fitting set (*A*_*st*_) and the three-center integrals between the fitting functions and the density overlap (*I*_*s*,μ*ν*_^(TT)^), and reduces the formal computational cost from O(N^4^) to O(N^3^).

We also extended the strategy to the exchange-correlation term,^93^ following the method introduced by Köster et al. in auxiliary nonrelativistic density functional theory.^99^ The approximated exchange-correlation matrix contribution to the DKS matrix is given by the expression
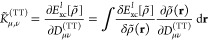
23where the functional derivative defines the exchange-correlation potential,  = , in which the fitted density is used. For the fitted electronic density obtained by the variational Coulomb fitting scheme, one has a convenient expression for the partial derivative, respect to the density matrix elements:
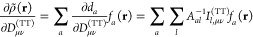
24where we use the three-index coulomb repulsion integrals, *I*_*l*,μ*ν*_^(TT)^, and elements of the inverse of coulomb interaction matrix, **A**, between auxiliary basis sets. Now substituting eq 24 into eq 23 and integrating, we obtain the approximated expression for the exchange-correlation matrix elements in a very simple form:
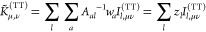
25with *z*_*l*_ being the elements of the vector solution of
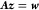
26where the vector ***w*** is the projection of the exchange-correlation potential (*ṽ*_xc_^(l)^[ρ̃](***r***)) on the fitting functions
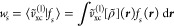
27The elements of the vector **w**, which involve integrals of the exchange-correlation potential, are computed numerically by the integration scheme already implemented in the code.^100^ Once the vectors ***d*** and ***z*** have been worked out, the Coulomb and the exchange-correlation contributions to the DKS matrix can be evaluated in a single step, in terms of three-center two-electron integrals *I*_*s*,μ*ν*_^(TT)^ = ⟨*f*_*s*_ ρ_μ*ν*_^(TT)^⟩:
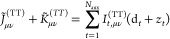
28

A key idea of this work has been to extend this scheme also to include the embedding potential contribution, *v*_emb_^I^[ρ_I_, ρ_II_](***r***). Indeed, the evaluation of the embedding potential matrix representation in G-spinors (*Ṽ*_*μν*_^*emb*(*TT*)^) can strictly follow the same procedure employed above for the exchange-correlation term and it reads as
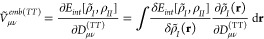
29where the functional derivative () defines the embedding potential, *v*_emb_^I^[ρ̃_*I*_, ρ_*II*_](**r**) (see eq 6), in which the fitted density of the active system is used. Using eq 24 in eq 29 and integrating, we have that the matrix elements, *Ṽ*_μ*ν*_^*emb*(TT)^, can be expressed as a linear combination of the three-index coulomb repulsion integrals as
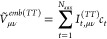
30The expansion coefficients (*c*_*t*_) are the elements of the vector ***c***, solution of the linear system
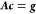
31and the vector ***g*** is the projection of the embedding potential, *v*_emb_^I^[ρ̃_I_, ρ_II_](***r***), on the fitting functions
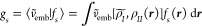
32The elements of the vector ***g*** are computed numerically using a suitable integration grid (further details of the present implementation will be given in the next section). The embedding potential contribution can be evaluated in a single step together with the Coulomb and the exchange-correlation ones, see eq 33, and finally added to the DKS matrix.
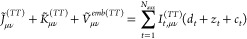
33

This procedure presents significant advantages respect to the direct matrix elements evaluation which needs the numerical integration of the embedding potential over the spinor basis set amplitude [i.e., *V*_*μν*_^*emb*(*TT*)^ = ∫ *v*_emb_(***r***) ρ_*μν*_^(*TT*)^(***r***)*d****r*** ], as is typically also employed in the four-component relativistic implementation of the FDE schemes.^26^ In particular, the cost of the numerical integration step, which dominates the computational burden, is significantly reduced and presents a lower scaling as the size of the active system increases. The latter scales as *N*_*b*_^2^ · *N*_*gridpoints*_, where *N*_*b*_ is the number of G-spinor basis functions and *N*_*gridpoints*_ is the number of grid points, while using the auxiliary fitting scheme presented above, the scaling is reduced to *N*^*aux*^ · *N*_*gridpoints*_, where *N*^*aux*^ is the number of fitting functions. The solution of the linear system in eq 31, which is also required here, scales as (*N*^*aux*^)^3^ but with a very small prefactor and, as we will show for a large set of molecular systems of increasing size, its actual contribution to the total elapsed time remains negligible (see Section 4). We mention that the scheme applied here to the evaluation of the FDE potential contribution is fully consistent with the auxiliary density functional theory^101^ and it can be efficiently employed, without significant modifications, in the nonrelativistic DFT implementations. Furthermore, in our specific case, an important aspect for the computational efficiency arises from the use of an auxiliary fitting basis set of primitive HGTFs. Indeed, they are grouped together in sets sharing the same exponents.^91^ In particular, each set is defined so that a specified auxiliary function of a given angular momentum is associated with all the corresponding functions of smaller angular momentum sharing the same exponent. This allows us to use the polynomial Hermite recurrence relations both in the analytical evaluation of the two-electron integrals for the Coulomb term and in the numerical representation of fitting basis functions used in the exchange-correlation and embedding potential contributions. This further reduces the burden of the expensive operation of evaluating large numbers of Gaussian exponents at each grid point.^93^

## 3 A DKS-in-DFT FDE implementation: the PyBerthaEmbed code

In this section we outline the computational strategy we adopted to implement the DKS-in-DFT FDE scheme. The developed Python program **pyberthemeb.py** and the related module (**pyembmod**) are freely available under GPLv3 license at ref (102).

**Figure 1:**
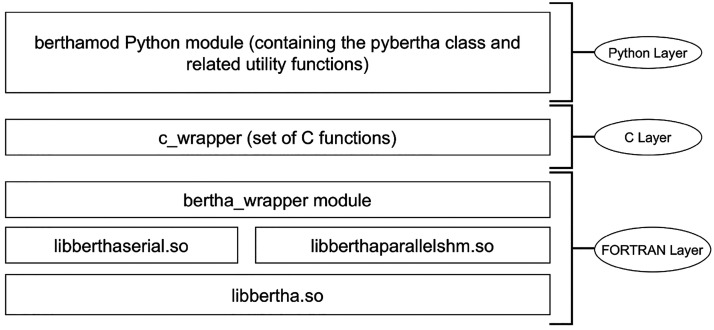
An overview of the BERTHA software’s layers.

Before addressing the FDE implementation, we present a brief outline the new Python^66^ API that has been recently implemented and that contributed to improve both the usability and interoperability of the BERTHA code.^74−76^ All these new features have been extensively employed in the workflow design and implementation of the DKS-in-DFT FDE method (see next section). In Figure 1 we outline the fundamental structure of BERTHA. All the basic kernel functions written in FORTRAN are now collected in a single Shared Object (SO) (i.e, **libertha.so**). Alongside there are two other SO libraries: **libberthaserial.so** capable of performing both the serial and parallel OpenMP based^103^ runs, and **libberthaparalleshm.so** containing all the functions needed to perform MPI^104^ based parallel computations where also the memory burden is distributed among the processes.

We also implemented a FORTRAN module, named **bertha**_**wrapper**, containing a class implementing all the methods needed to access to all the basic quantities, such as energy, density, DKS and overlap matrices and other. The same FORTRAN module (i.e., **bertha**_**wrapper**) is used to perform all the basic operations such as **bertha**_**init** to perform all the memory allocations, **bertha**_**main** to run the main SCF iterations, and **bertha**_**finalize** to free all the allocated memory, and more. Finally the main PyBERTHA^67^ module has been developed using the **ctypes** Python module. This module provides the C-compatible data types, and allows calling functions collected in shared libraries. In order to simplify the direct interlanguage communication between Python and FORTRAN, we implemented a simple C layer called **c**_**wrapper**, also summarized in Figure 1. This Python API to BERTHA has been described in detail in refs (74, 76) and in the present work has been further extended with new methods which allow us to extract all those quantities necessary for the DKS-in-DFT FDE implementation (e.g., the method **bertha**_**get**_**density**_**on**_**grid()** used to extract the values of the fitted electron density on a grid). All the new methods have been efficiently parallelized using OpenMP.^103^ All details and computational efficiency will be given in the next sections.

Thanks to our development of PyBERTHA, the implementation of the DKS-in-DFT FDE method (PyBERTHAembed software) resulted in being straightforward and relatively simple. We have been able to handle different aspects and quantities involved in the workflow also coming from different codes as a single unit and in a common framework based on Python. The newly developed code is composed of two main modules: the **pyembmod** one, which allows one to manage the important quantities for the FDE implementation, and the **pyberthamod** module.^67^

Specifically, the **pyemb** class inside the **pyembmod** module allows one to well isolate all the FDE data and operations increasing the level of abstraction. The module is used to manage all the required quantities for the generation of the embedding potential, that is *v*_emb_[ρ̃_I_, ρ_II_(***r***)]. It has been engineered in a such manner that all details of the FDE low-lying implementation will be completely transparent from the PyBERTHA side. This has the advantage that all future developments and/or integration of the FDE scheme (g.e. using DKS theory also for the environment DKS-in-DKS FDE) will not affect the PyBERTHAembed code, i.e., it will remain completely unchanged. In particular, in this first version, the **py embmod** module can handle the basic procedures previously implemented in the Psi4-rt-PyEmbed software, which are based on the use of PyADF,^30,68^ PyEmbed module,^69,70^ and the XCFun library^71,72^ to evaluate nonadditive exchange-correlation and kinetic energy contributions on user-defined integration grids. This approach gave us both the advantages of the code reusability and, even more importantly, a DFT-in-DFT FDE reference implementation in which we can have precise control over all those details and parameters from which a FDE scheme is dependent on (i.e., algorithms, numerical grid definition, quantum chemistry packages used to determine electronic density and Coulomb potential of the environment, basis sets, exchange-correlation functionals, etc.). This has clearly made the debugging phase in the development of PyBERTHAembed software straightforward. The **pyberthemeb.py** code is freely available at ref (102), and the most important part has been also described in detail in the SI (see Algorithm 1 and its tutorial-like description in the SI).

**Figure 2:**
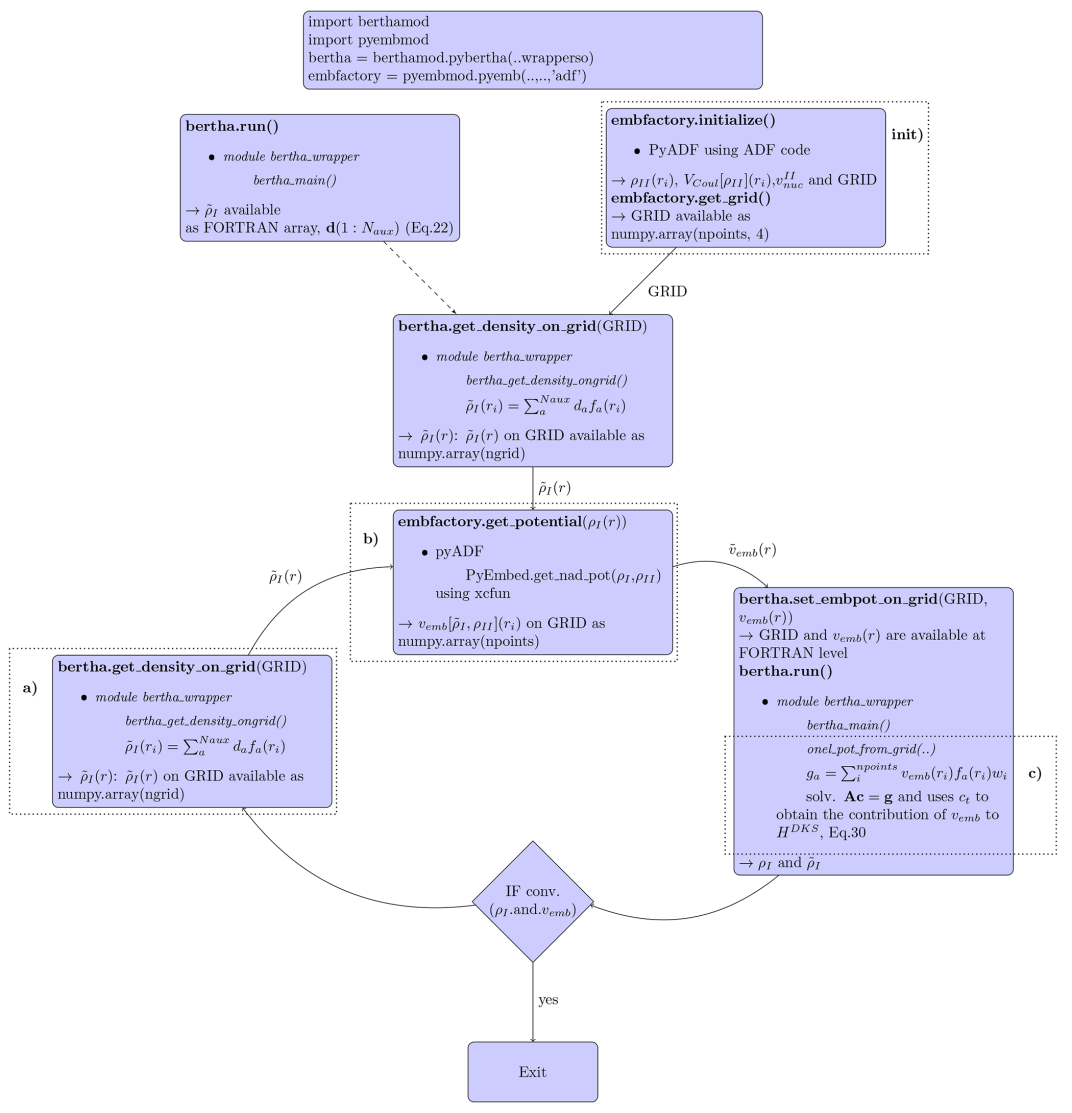
Working flowchart of the Pyberthemeb. The dashed boxes highlight the main tasks related with the FDE implementation: (/init) the density and electrostatic potential of the environment are obtained as grid functions in the out-of-loop section; (a) numerical representation of ρ̃(*r*) on the grid; (b) PyEmbed classes are used to calculate the embedding potential; and (c) projection of the embedding potential onto fitting basis functions.

In Figure 2, we present a workflow that emphasizes the interoperability between different tasks and modules or programs involved including the layers where the actual computations are performed. This schematic picture also highlights how the key quantities, which are required to implement the DKS-in-DFT FDE scheme, have different representations along the computation. As an example, we focus on the electron density of the active system, ρ̃_*I*_(***r***). This quantity is evaluated at the DKS level of theory activated by the **bertha.run()** method within the PyBERTHAembed program. The actual calculation is done within the FORTRAN layer. At this level, ρ̃_*I*_(***r***) is represented in terms of the expansion coefficients of auxiliary fitting functions (***d***; see eq 18) and is stored as a FORTRAN array (of dimension *N*_aux_). However, this representation is not useful itself for the evaluation of the embedding potential. Indeed, its evaluation and, in particular, the nonadditive contribution requires that ρ̃_*I*_(***r***) is represented on a grid. Thus, within **pyberthemeb.py**, the numerical grid (GRID) evaluated within PyADF (see panel init) is made available as **numpy.array** and via the **bertha.get**_**density**_**on**_**grid()** method is made accessible to the FORTRAN layer (**bertha**_**wrapper** module), and stored as a FORTRAN array of dimension *npoints*. The calculation of the numerical representation of ρ̃_*I*_(***r***) on the grid is done efficiently in FORTRAN (see in Figure 2, panel a) and the latter is accessible within **pyberthemeb.py** as a **numpy.array**. Analogously, different representations are also used for the embedding potential along the workflow. Note that all quantities accessible from **pyberthemeb.py**, namely, ρ̃_*I*_(*r*_*k*_), *v*^*emb*^(*r*_*k*_) and GRID (labeling the arrows in figure), are defined as **numpy.array** that can be easily manipulated within a Python source code. The computational steps that involve BERTHA are instead implemented in FORTRAN panels (a) and (c) in Figure 2) and have been efficiently parallelized using OpenMP.

## 4 Results and Discussion

In the present section, we report a series of numerical results mainly devoted to assess the correctness of our new implementation of the DKS-in-DFT FDE scheme. In addition, we are also reporting the computational cost and scalability, with respect to the size of both the active and the embedding system. Finally, we will present an application to a series of heavy (Rn) and super-heavy elements (Cn, Fl, Og) confined into a C_60_ cage.

A dataset collection of computational results including numerical data, parameters, and job input instructions used is available and can be freely accessed at the Zenodo repository (see ref (105)).

### 4.1 Initial Validation and Numerical Stability: H_2_O-NH_3_

As already mentioned, in this first version of the **pyembmod** module, we include the basic procedures previously implemented in the Psi4-rt-PyEmbed code, which are based on the use of PyADF^30,68^ and of the PyEmbed module.^69,106^

This positions us in an ideal framework of having a reference nonrelativistic DFT-in-DFT FDE implementation, where we can have precise control over all those details, and parameters, upon which a FDE calculation is dependent. Thus, for the sake of a direct comparison, we selected a simple molecular complex—namely, the H_2_O-NH_3_ adduct—for which the relativistic effects are expected to be negligible. For this system, we can safely compare directly the numerical results of the DKS-in-DFT FDE method, implemented here, with respect to those obtained using the DFT-in-DFT FDE scheme in the Psi4-rt-PyEmbed code.^31^ In the adduct, the water molecule is the active system that is bound to an ammonia molecule, which instead plays the role of the embedding environment. The molecular structure of the adduct is reported in Table S.1 in the SI. The effect of the environment (ammonia) on the active system (water) has been evaluated by comparing the dipole moment components and diagonal elements of the polarizability tensor (α_*xx*_, α_*yy*_, and α_*zz*_) of the isolated (Free) respect to the embedded (Emb) water.

The full numerical results are reported in the SI (see Table S2 and related comments) and they show an evident quantitative agreement between the two implementations. We mention that we also performed the calculations increasing the speed of light by 1 order of magnitude (i.e., *c* = 1370.36 a.u.) to approximate the nonrelativistic limit and, as expected, we obtain almost indistinguishable results (see Table S3 in the SI). All these findings make us confident that our implementation is both numerically stable and correct.

As we have extensively described in the previous section, our implementation strongly benefits from the use of auxiliary fitting functions, both in the definition of the embedding potential and as intermediate quantities to obtain its G-spinor matrix representation. Thus, it appears mandatory to investigate the impact of the quality of the density fitting basis set on the final results of DKS-in-DFT FDE calculations. In addition to the limit auxiliary fitting basis set (A4_spdfg_) employed above for the validation calculation, we generated five fitting basis sets (A2_s_, A2_sp_, A2_spd_, A2_spdfg_, and A3_spdfg_) of increasing accuracy. We have adopted a procedure that is strictly related to that proposed by Köster et al. and employed in Demon2K code (see the appendix of ref (107)). All the fitting basis sets are explicitly reported in the SI, while the results are reported in Table 1. In the table, we also show the absolute error in the Coulomb energy (Δ*E*_*J*_), which is the quantity that is variationally optimized in the fitting procedure and typically regarded as its quality index. This numerical test shows that the use of density fitting does not introduce any significant instability in the DKS calculation of the active system, also in the presence of the embedding potential. The Δ*E*_*J*_ values are showing a convergent trend of both the dipole moment components and the polarizability when the quality of the fitting basis set is increased. The fitting basis sets A2_s+_ and A2_sp+_, bearing only s- and p-type Hermite Gaussian functions, have values of Δ*E*_*J*_ larger than 1 mEh and are clearly inadequate to reproduce the reference results. Very accurate results can already be obtained starting from the A2_spd_ auxiliary basis set (i.e., 163 functions for the water molecule). It is interesting to note that the Δ*E*_*J*_ associated with this basis set is of the same order of magnitude of that typically required (0.1 mEh per atom) in standard calculations, based on density fitting without including FDE. Thus, these preliminary results suggest that the variational density fitting scheme can safely be applied in the implementation of DKS-in-DFT method without jeopardizing its accuracy.

**Table 1:** Dipole Moment (Components μ_*x*_, μ_*y*_, μ_*z*_ and Module |μ|) and Dipole Polarizability (Tensor Diagonal Components α_*xx*_, α_*yy*_, α_*zz*_ and Isotropic Contribution α_iso_) of the Embedded Water Molecule (Water-Ammonia System)^a^

	A2_s_	A2_sp_	A2_spd_	A2_spdfg_	A3_spdfg_	A4_spdfg_
*N*_aux_	(19)	(67)	(163)	(338)	(403)	(544)
μ_*x*_	–0.49845	–0.50555	–0.49264	–0.49250	–0.49261	–0.49267
μ_*y*_	–0.65654	–0.64784	–0.64804	–0.64415	–0.64445	–0.64429
μ_*z*_	–0.00034	–0.00051	–0.00024	–0.00028	–0.00028	–0.00028
|μ|	0.824322	0.82175	0.81404	0.81086	0.81116	0.81107
α_*xx*_	8.46	7.95	7.96	7.96	7.96	7.96
α_*yy*_	7.19	8.12	7.95	7.95	7.95	7.95
α_*zz*_	3.90	6.08	5.80	5.81	5.81	5.82
α_*iso*_	6.52	7.38	7.24	7.24	7.24	7.24
Δ*E*_*J*_	1.51 × 10^–2^	4.47 × 10^–3^	2.8 × 10^–4^	4.8 × 10^–6^	1.9 × 10^–6^	5.0 × 10^–7^

a Data have been obtained with our new PyBERTHAembed implementation (using a G-spinor basis functions derived from the cc-pvtz-decon basis) and several auxiliary density fitting basis sets (A2_s_, A2_sp_, A2_spd_, A2_spdfg_, A3_spdfg_, and A4_spdfg_). The sizes of different fitting basis sets (N_aux_) are reported in parentheses. Δ*E*_*J*_ is the absolute error on the Coulomb energy due to the density fitting. The diagonal components of the dipole polarizability tensor have been calculated with a finite field approach, using an external electric field of 0.001. All numerical data are reported in atomic units (a.u.). See text for the fitting basis set definition and further details.

### 4.2 Computational Efficiency: Gold Clusters in Water

It is interesting now to put forward some assessments on the computational efficiency of our DKS-in-DFT FDE implementation, together with its scaling properties in terms of time statistics and memory usage. This analysis will give us a detailed overview of the computational burden, and possible bottlenecks, along the relatively complex workflow we implemented (using different quantum chemistry packages and programming languages). Furthermore, it will be a solid starting point for future optimizations and developments (e.g., DKS-in-DKS or coupled real time DKS-in-DKS). As a test case, we have chosen a series of gold clusters (Au_2_, Au_4_, Au_8_) embedded using an increasing number (5, 10, 20, 40, and 80) of water molecules. In all cases, for Au, the large component of the G-spinor basis set was generated by uncontracting double-ζ quality Dyall’s basis sets^108−110^ augmented with the related polarization and correlating functions (24s19p12d9f1g), while the corresponding small component basis was generated using the restricted kinetic balance relation. For the water molecules of the environment, we used the DZ Slater-type set from the ADF library.^111^ The supermolecular grid defined in PyADF, corresponding to an integration parameter of 4 in the ADF package, has been used. The BLYP^112,113^ exchange-correlation functional is used for the ground-state calculation of the embedding system, while the Thomas–Fermi and LDA functionals^114,115^ have been employed for the nonadditive kinetic and nonadditive exchange-correlation potential, respectively. The structures of clusters have been obtained by simple geometry optimization using the ADF code with a small basis set (DZ) and ZORA Hamiltonian, and they are available in ref (105).

The results are reported in Tables 2 and 3, where, together with the total elapsed time (*t*^d^) for each SCF iteration, including the FDE contribution, we also partition between different tasks related with the FDE implementation, namely, (a) numerical representation of an active system fitted density on grid; (b) calculation of the nonadditive terms of embedding potential by PyADF (with the PyEmbed class); (c) projection of the embedding potential onto fitting basis functions. In these tables, we also report the maximum memory usage for the SCF procedure (“Mem”), the number of points of grid and the timing for the “init” phase which involves: the evaluation of the ground-state electronic density of the environmental together with the associated Coulomb potential, and their mapping on the numerical grid. We recall that the electron density of the environment is kept frozen; thus, this initial step is done once at the beginning of the procedure. All tasks are also highlighted (using the same labeling: a, b, c and init) in Figure 2.

**Table 2:** Elapsed Real Time

system	*t*^a^ (s)	*t*^b^ (s)	*t*^c^ (s)	*t*^d^ (s)	memory (MB)	grid points	init embfactory
Au_2_(H_2_O)_10_	1.47	2.74	1.48	42.68	1165	213248	138.9 (8.8)
Au_4_(H_2_O)_10_	3.06	2.84	3.09	260.16	2164	221824	127.9 (9.0)
Au_8_(H_2_O)_10_	6.62	3.05	6.70	1849.90	7572	237824	154.7 (9.8)

a Fitted density on grid.

b Calculation of the nonadditive terms of embedding potential by PyADF (with PyEmbed classes).

c Projection of the embedding potential onto fitting basis functions.

d Total time for a single DKS self-consistent field interaction. All the calculations have been performed on a Dual Intel Xeon CPU E5–2684 v4 running at 2.10 GHz, equipped with 251 GiB of RAM. We used the Intel Parallel Studio XE 2018^116^ to compile the FORTRAN code and Python 3.8.5 (from Anaconda, Inc.) and NumPy version 1.19.2 for the Python code. We used PyADF^30,68^ as recently ported to Python3,^65^ ADF (version 2019.307) for the core DFT calculations of the environment and *XCFun* library (version 1.99).^71,72,117^

**Table 3:** Elapsed real time

system	*t*^a^ (s)	*t*^b^ (s)	*t*^c^ (s)	*t*^d^ (s)	memory (Mb)	grid points	init embfactory
Au_4_(H_2_O)_5_	1.97	1.84	1.99	257.70	2137	143232	102.3 (5.9)
Au_4_(H_2_O)_10_	3.06	2.84	3.09	260.16	2164	221824	127.9 (9.0)
Au_4_(H_2_O)_20_	5.04	4.71	5.07	260.44	2225	366336	215.4 (14.8)
Au_4_(H_2_O)_40_	8.69	8.09	8.71	270.19	2331	630144	641.0 (25.8)
Au_4_(H_2_O)_80_	16.27	15.19	16.40	295.65	2354	1184896	2600.1 (47.8)

a Fitted density on grid.

b Calculation of the nonadditive terms of embedding potential by PyADF (with PyEmbed classes).

c Projection of the embedding potential onto fitting basis functions.

d Total time for a single DKS self-consistent field interaction. See the caption of Table 2 for the computational details.

As a general remark, we may state that the FDE contribution to the total time is relatively small. By increasing the size of the active system (Au_2_, Au_4_, and Au_8_), and keeping the environment fixed (using ten water molecules; see Table 3), the relative impact of the FDE computational phase decreases. It passes from 13.3% for Au_2_(H_2_O)_10_ to 0.9% for Au_8_(H_2_O)_10_. This may be expected since tasks (a), (b), and (c) have a more favorable scaling than the DKS calculation (i.e., *O*(*N*^3^)). The computational cost for steps (a) and (c) is proportional to the product *N*^aux^ · *N*_gridpoints_, where *N*^aux^ is the total number of the auxiliary fitting functions in the active system, and *N*_gridpoints_ total number of grid points. Thus, the computational cost should scale as *O*(*N*^2^) (with *N* being the dimension of the active system). The actual scaling is much lower (i.e., slightly higher than *O*(*N*)), mainly because the total grid points are largely dominated by the environmental system (see the number of grid points, *N*_gridpoints_, as reported in Table 2). Concerning step (b) and considering the fact that the environment is maintained fixed, it scales, as expected, linearly with number of points of the grid (*N*_gridpoints_). The maximum use of memory, during the entire DKS-in-DFT FDE procedure, increases with respect to the number of Au atoms being N^1.7^, which is close to the theoretical value N^2^.

When we fix the active system (Au_4_) increasing instead the size of the environment, see Table 3, the relative computational cost to include the embedding passes from 2.2%, in the case of Au_4_@(H_2_O)_5_, to 16.1% for Au_4_@(H_2_O)_80_. In this case, all tasks associated with the FDE procedure (a, b, and c) have a computational burden that increases linearly with the size of the environment (and the number of total grid points; see Figure S1 in the SI), while the maximum memory usage during the SCF procedure is almost independent from the number of water molecules in environment, as only a slight increase can be observed.

As already mentioned in the previous sections, we have recently developed an OpenMP parallel version of BERTHA, which can be easily used directly via the Python API.^75^ This only requires the **berthamod** module, which refers to the shared object **libberthaserial.so**, to be compiled with OpenMP flag set. Thus, here we have extended the OpenMP parallelization to those steps of the FDE procedure in which the BERTHA code is directly involved, namely, steps (a) and (c) (see above). The results are given for the Au_4_(H_2_O)_80_ system and are reported in Table 4. These steps have been efficiently parallelized, and we are able to achieve a speedup of 31.1 and 29.8 using 32 threads, respectively, for steps (a) and (d). Noteworthy, for this parallel implementation, the FDE phase is ∼45% of the total elapsed time and is dominated by the computation task that remains a serial part. Indeed, using 32 threads, the task described by step (b) takes 15.20 s of the total 35.8 s necessary for each SCF iteration. This task, which is related to the generation of the nonadditive kinetic and exchange-correlation on grid, is currently performed by the PyEmbed component in PyADF. Regarding the memory usage, in our OpenMP implementation, we observe a linear growth of memory usage, with respect to the number of the employed threads. This is somehow expected due to the obvious data replication in the OpenMP implementation. Despite the fact that one may expect that there may be room for further optimization, we note that, even in the current version, the implementation is not memory-bound. In the case of 32 threads, we found a maximum memory usage of ∼11 GiB, which demonstrates that such types of calculations, and even larger ones, can be routinely performed on the current multicore architectures, which may easily achieve 64 to 128 cores and 512 to 1024 GiB per node.

**Table 4:** Elapsed Real Time for the Au_4_(H_2_O)_80_ System

number of threads	*t*^a^ (s)	*t*^b^ (s)	*t*^c^ (s)	*t*^d^ (s)	memory (Mb)	init embfactory
1	16.16	15.16	16.40	291.06	2343	2634.4 (48.5)
2	8.12	15.23	8.30	158.20	2638	2633.1 (48.8)
4	4.07	15.23	4.11	91.10	3210	2631.4 (48.5)
8	2.03	15.32	2.06	60.48	4377	2655.7 (48.4)
16	1.04	15.23	1.10	43.67	6632	2603.4 (48.5)
32	0.52	15.20	0.55	35.80	11020	2601.4 (49.1)

a Fitted density on grid.

b Calculation of the nonadditive terms of embedding potential by PyADF (with PyEmbed classes).

c Projection of the embedding potential onto fitting basis functions.

d Total time for a single DKS self-consistent field interaction. All of the running times have been obtained using the dynamic schedule in OpenMP, see the caption of Table 2 for the computational details.

### 4.3 The Generation of Atom-Endohedral Fullerenes Model Potentials

We conclude our work by showcasing how we can leverage our FDE implementation to determine fullerene-atom model potentials that are applicable for species across the periodic table.

Over the past decades, it has been recognized that fullerenes can serve as containers for other, smaller species.^118^ As such, there has been considerable interest in understanding how such smaller species behave under confinement, both from a fundamental point of view as well as due to possible technological applications we mention: the potential use as seed materials in solid-state quantum computation,^119^ and the use as agents for improving the superconducting ability of materials.^120^

From a more fundamental perspective, the study of how atomic species behave under such confinement is a particularly active domain. With respect to the use of theoretical approaches, many studies have been reported that employed, in most cases, simple models of the C_60_ cage potential to represent the confinement potential.^121−123^ A model where only electrons of the guest atoms are considered while the *C*_60_ cage is modeled, in most cases, by a short-range attractive *V*_c_(*r*) spherical potential that is defined as follows:
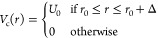
34where *r*_0_ = 5.8 a.u. and *U*_0_ = −0.30134 a.u. and Δ = 1.9 a.u represent the finite thickness of the spherical potential.^121^ Other model potentials have been proposed,^124^ as well as other approaches to avoid numerical instability related to the sharp form of those potentials.^125,126^ An alternative approach may be to start from the embedding potential generated in the FDE scheme, which is expected to be highly accurate and without artificial discontinuities. In the following, we propose a possibly general procedure to build model potentials for atomic calculations and, with this aim, we compare the results, in terms of HOMO–LUMO gap, for a set of heavy atoms, obtained using the Frozen Density Embedding (FDE) procedure respect to the simple spherical potential model (SPM) reported in eq 34. Practically we applied the FDE scheme to a set of neutral endohedral fullerenes A@C_60_ (A = Rn, Og, Fl, Cn), where the atom A (i.e., active system) is embedded in a fullerene (i.e., environment) and placed at the exact center of the C_60_. Finally, by comparing the embedding potential (EMBP) and its spherical average with respect to SPM, we propose a simple numerical recipe that can be used within the FDE scheme to possibly extract more accurate potentials to be tested in atomic calculations.

Before proceeding in the comparison of different models, we have investigated the ability of FDE to capture environmental effects analyzing the orbital energies differences, with respect to the standard (supramolecular) DFT calculations and we have also estimated the effect of the mutual polarization between fragments which is neglected in our FDE implementation within PyBerthaEmbed. All these test calculations have been carried out on Rn@C_60_ at scalar ZORA level using the ADF code. The results are summarized in the SI (Figures S2 and S3). Despite the orbital energy shift is (in particular for inner electrons) strongly dependent on the specific model potential employed in ADF code for implementing the ZORA Hamiltonian (see also the caption of Figure S2 for details), as a consequence of the Gauge dependence the ZORA equation,^127^ there is a substantial agreement between the FDE and supramolecular calculations. The FDE approach yields a overall slightly larger orbital energy shifts (the free Rn has been taken as reference), that are nevertheless very homogeneous across different orbitals, see Figure S2. The agreement between the FDE scheme and the supermolecular calculation becomes even more stringent as one also includes the mutual polarization effects between fragments using the freeze-and-thaw procedure (see Figure S3). This latter finding clearly suggests that it will be important in the future to extend the PyBerthaEmbed code and to include such polarization effects which cannot be totally neglected if one desires to describe these phenomena with high accuracy.

All calculations, reported in the following, have been performed with the PyBerthaEmbed code, using a DKS Hamiltonian and a basis set for the active system (i.e., A = Rn, Og, Fl, Cn) generated by uncontracting triple−ζ quality Dyall’s basis sets^109,110,128,129^ augmented with the related polarization and correlating functions. Final basis set schemes are as follows: Cn (32s29p20d14f7g2h), Rn (31s27p18d12f4g1h), and Fl and Og (31s30p21d14f6g2h). For all the elements, we used auxiliary basis sets already employed in ref (130). and are explicitly reported in the SI. While for the environment (i.e., the C_60_), computed using the ADF code, we use the TZP basis set and nonrelativistic Hamiltonian. In both cases, we use the BLYP^112,113^ exchange-correlation functional, while for the nonadditive kinetic and nonadditive exchange-correlation terms in the generation of the embedding potential, the Thomas–Fermi and LDA functionals are used, respectively.

**Figure 3:**
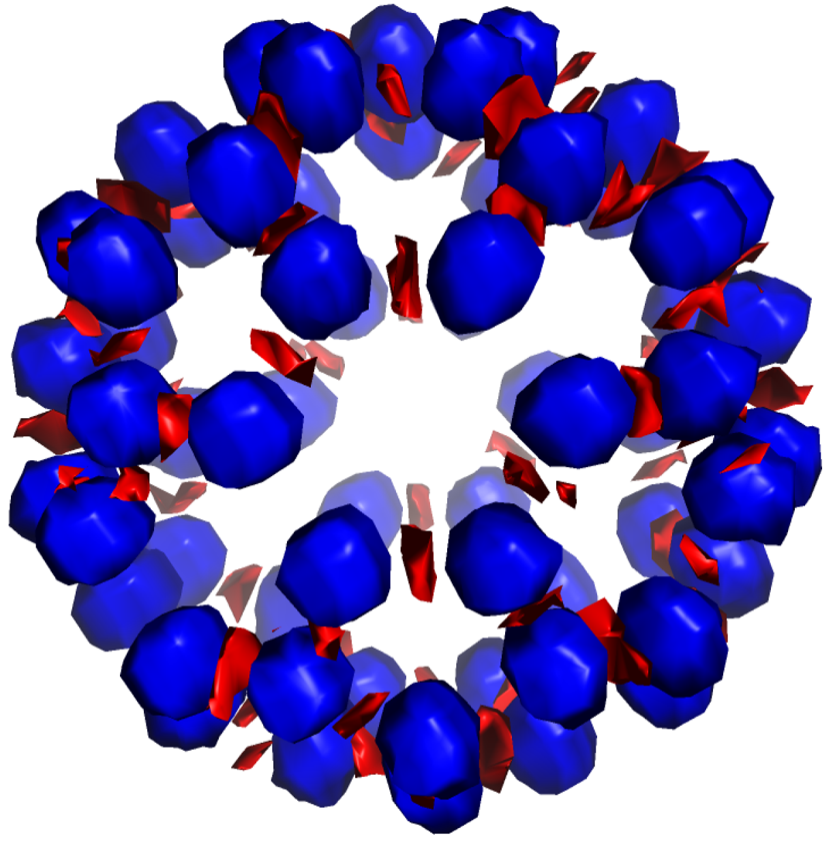
Embedding potential (EMBP) in blue (negative) and red (positive), computed for the Rn@C_60_ system. We report the contour plot at ±0.3 a.u. It is important to underlined as the plotted values are the result of a nearest-neighbor interpolation performed starting from the original nonhomogeneous ADF grid.

**Figure 4:**
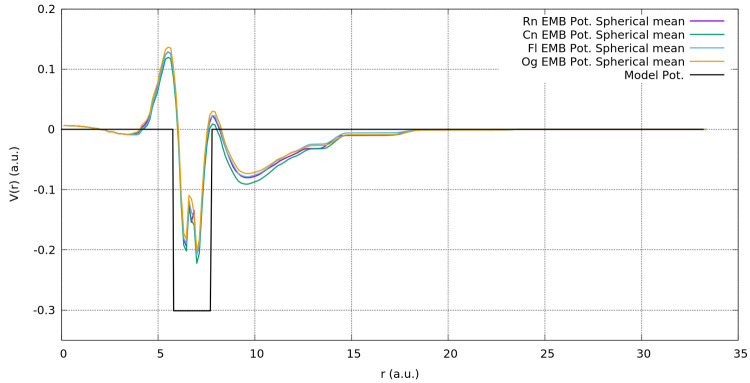
Spherical average of the Rn, Og, Cn, and Fl embedding potential together with the simple short-range attractive spherical potential, as reported in eq 34.

Figure 3 reports the embedding potential (EMBP) of the Rn@C_60_ system. The EMBP shows positive values centered at nuclei positions, and negative values located in correspondence of the bonds. As one may expect, the EMBP potential, while maintaining an overall spherical shape, is clearly different, with respect to a simple short-range attractive *V*_c_(*r*) spherical potential model (SPM). Indeed, if we consider the spherical average of the EMBP (see the SI for details on the spherical average procedure employed) extracted for the various A@C_60_ systems, as reported in Figure 4; while the EMBP seems to detect the same short-range attractive values, surely it shows a more complex radial structure. The spherical average of the EMBP shows a positive repulsive value immediately before the inner C_60_ surface and, maybe more importantly, never completely goes to zero, not even at the center of the fullerene where atom A is placed. It is eye-catching that these averaged embedding potentials generated for the different active systems are very similar and are almost identical at small values of *r*. We will revisit this interesting point later in this section.

**Figure 5:**
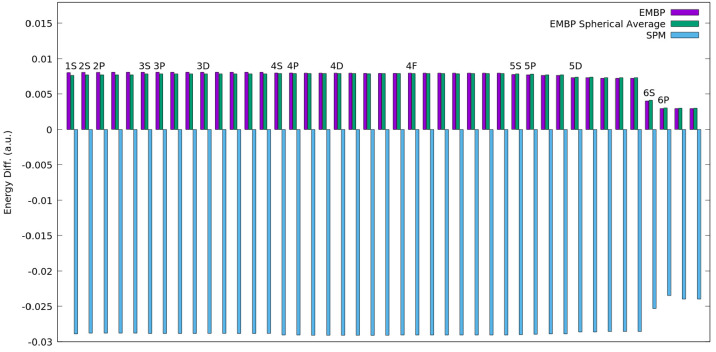
Differences in orbital energies, with respect to the isolated Rn atom for all occupied orbitals, for the SPM model, the EMBP, and the spherical average of the EMBP.

The numerical results (see Table 5) reported in terms of HOMO–LUMO gap for SPM are quite different, with respect to the results obtained using the full FDE procedure (see columns 2 and 3 of Table 5, respectively). Indeed, as we mentioned, the overall shape of the EMBP is quite different, with respect to a simple spherical one; see Figure 3. Nevertheless, is interesting to note tht the spherical average seems to work well. Comparing the results obtained from the full FDE procedure, with respect to the ones computed using a model potential that is the spherical average of the EMBP (columns 3 and 4, respectively, in Table 5), one can easily note that the spherical average is able to well reproduce the electronic structures of the active system (i.e., the central atom) including HOMO–LUMO gaps values with an error that is generally <1%. Similar conclusions can be drawn looking at Figure 5, where we report instead all the differences in orbital energies, with respect to the isolated Rn atom for all the occupied orbitals. Once again, both the EMBP (i.e., the FDE procedure) and its spherical average lead to similar results. Instead, by using the simple spherical model (SPM), the energy shift is always the opposite. The large negative values of the orbital energies found for the SPM potential is somehow unexpected. Indeed, the spinor solution of four-component Dirac Hamiltonian must be gauge invariant, regardless of whether one adds a constant to the Hamiltonian; thus, for the core orbitals and, in particular, for 1s spinors, one may expect that the energy shift would be mostly determined by the value of the embedding potential at the central nuclei position. Meanwhile, the latter is exactly what we have found in the case of the spherical average of the EMBP and of the FDE potential, which produce a 1s energy shift (see Table S4 in the SI), almost identical to the value of the embedding potential at the C_60_ center (i.e., the spherical average of EMBP at the fullerene center is 0.006230 a.u.), the same cannot be observed for the SPM potential. The latter is indeed largely negative (−0.030386 a.u.), which is clearly inconsistent with the zero value that the SPM potential assumes at the C_60_ center (see eq 34). A deep understanding of this issue would require a systematic numerical analysis, which is beyond the purpose of this work; however, the fact that a sharp shape of a potential like the SPM may introduce a certain numerical instability has been already reported in the literature (see, for instance, ref (126)). Moreover, it is interesting to note that regardless of whether we use a multisteps model potential, more similar to the EMBP spherical average, the inconsistency is significantly reduced, see Table S4 and Figure S4 in the SI.

**Table 5:** HOMO-LUMO Gap Energies

	Gap Energy (a.u.)			
			Spherical Average	
atom	SPM	FDE C_60_	EMBP	Rn-based EMBP
Rn	0.118680	0.209643	0.207990	–
Cn	0.055807	0.144685	0.144693	0.147514
Fl	0.072251	0.110471	0.110467	0.108101
Og	0.148040	0.139842	0.139300	0.134420

As already noted, in Figure 4, the embedding potentials generated for the entire series of systems are very similar, thus one may expect a certain transferability of the embedding potential between different active systems. By evaluating the spherical average of the EMBP for the Rn@C_60_ system and using this single model potential, we have been able to quantitatively well reproduce the HOMO–LUMO gap for all the other A@C_60_ systems. The numerical results are reported in column 5 of Table 5, where we see that the HOMO–LUMO gaps for all the atoms have an error that is always <4%, using a Rn-based EMBP spherical average. This finding demonstrates that, for this specific class of systems, the FDE potential and its spherical average appear to be easily transferable.

**Figure 6:**
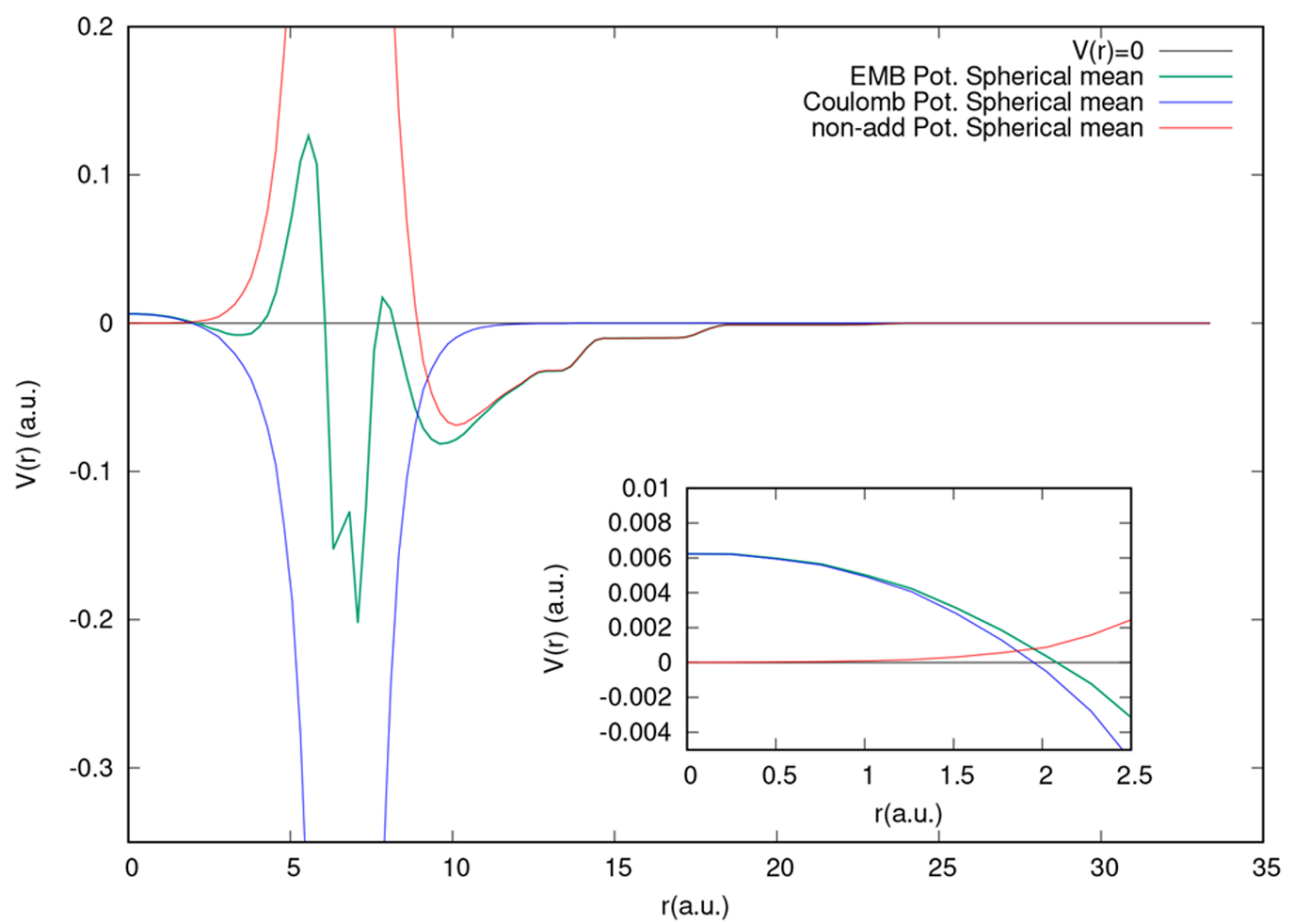
Separation of the EMBP spherical average into its constituents: the constant Coulomb potential and the nonadditive (exchange-correlation plus kinetic energy) terms. Data are for the Rn@C_60_ system.

In Figure 6, we report the spherical average of EMBP disentangled into its constituents: (i) the Coulomb potential, based only on the fixed electron density of the environment, and (ii) the sum of nonadditive (exchange-correlation and kinetic energy) terms. Data are reported for the Rn@C_60_ system. The embedding potential is clearly dominated at small distances (0 < *r* < 1.7 a.u.) by the fixed Coulomb potential of C_60_, while it is essentially determined by the nonadditive terms for large values of *r* (*r* > 10 a.u.). In the medium range, EMBP results from a large positive nonadditive terms contribution and a large negative Coulomb potential is generated by the C_60_ fragment, which has a tendency to cancel out and leads to the observed oscillating pattern. This analysis suggests that the observed good transferability of the embedding potential can be mainly associated with the fact that at short-range (which is the strong coupling region) it coincides with the fixed Coulomb potential and so it is independent of the active system. We mention that the Coulomb potential term would not be independent of the active system in case one includes the mutual polarization effects between fragments (using, for instance, freeze-and-thaw cycles and switching the rule of the active/embedding systems). In this case, the dependence on the active system may jeopardize the good EMBP transferability property observed above. Before concluding, it is interesting to investigate how the spherical average of the EMBP performs when one introduces modifications to the relative position of fragments, and the central atom is displaced from its the central position. In Table 6, we report the HOMO–LUMO gap of the Rn atom when placed off-center of the C_60_. Interestingly, the model potential obtained considering the spherical average of the EMBP is able to well reproduce the overall HOMO–LUMO gap decrease detected by the full FDE calculations. Nevertheless, the absolute difference, which also is reported in Table 6, shows, as one may expect, that the spherical model potential becomes evidently less accurate as the atom moves further away from the center (reaching a maximum difference of ∼5% when the atom is shifted more than 3 a.u., with respect to <1% when the atom is placed at the exact center of C_60_ (see Table 5)).

**Table 6:** HOMO-LUMO Gap Energies (a.u.) for the Rn Atom at Increasing Distances, along the *x* axis, from the Center of the C_60_

	Gap Energy (a.u.)		
Rn *x*-axis shift (a.u.)	FDE C_60_	EMBP (spherical average)	difference (%)
0.406664	0.208213	0.205551	1.29
0.813329	0.204787	0.200096	2.32
1.626658	0.195770	0.186477	4.86
3.253317	0.190614	0.181483	4.91

Although this study does not claim to be conclusive, the findings presented above let us clearly envision a practical approach to build model potential as a result of the FDE procedure.

## 5 Conclusions and Perspectives

Including environmental effects based on first-principles is of paramount importance in order to obtain an accurate description of molecular species in solution and in confined spaces. Among others, the frozen density embedding (FDE) density functional theory (DFT) represents an embedding scheme in which environmental effects are included by considering explicitly the environmental system by means of its “frozen” electron density. In the present paper, we reported our extension of the full 4-component relativistic Dirac–Kohn–Sham method, as implemented in the BERTHA code, to include environmental and confined effects with the FDE scheme (DKS-in-DFT FDE) using the PyADF framework. We described how its complex workflow associated with its implementation can be enormously facilitated by the fact that both BERTHA (PyBERTHA) and PyADF, with their Python API, they gave us an ideal framework of development. The recent development of the Psi4-rt-Embed code,^31^ which is also based on PyADF for FDE while using Psi4Numpy code for the active system, represented an ideal reference implementation to assess the correctness of our new DKS-in-DFT FDE implementation.

PyBERTHAembed uses the density fitting technique at the key points of the interface between PyBERTHA and PyADF. We showed that this results both in a very efficient numerical representation of the electron density of the active system and in a straightforward evaluation of the matrix representation in the relativistic G-spinor basis of the embedding potential.

The accuracy and numerical stability of this approach, also using different auxiliary fitting basis sets, has been demonstrated on the simple NH_3_–H_2_O system. We compared the dipole moment components and diagonal elements of the polarizability tensor of the isolated water molecule with respect to the embedded water (i.e., NH_3_–H_2_O system). We performed the calculations using both our DKS-in-DFT FDE implementation, as well as the previously implemented Psi4-rt-PyEmbed code. The numerical results shown an evident quantitative agreement between the two implementations. Indeed, both variations induced by the presence of the embedding system and the absolute values of both the dipole moments and polarizability show a good agreement. Noteworthy, independently by the basis set used, the differences are below 0.001 a.u. and 0.01 a.u. for the dipole moment components and for the polarizability tensor components, respectively.

We also evaluated the computational burden on a series of gold clusters (Au_*n*_, with *n* = 2, 4, 8) embedded into an increasing number of water molecules (5, 10, 20, 40, and 80 water molecules). We found that our implementation approximately scales linearly both with respect to the size of the frozen surrounding environment and the size of the active system. We efficiently parallelized, using OpenMP, two of the most demanding steps on our computation, that is the computation of the numerical representation of active system fitted density on grid, as well as the projection of the embedding potential onto fitting basis functions. The results reported show that we are capable of reaching a final speedup of 31.1 and 29.8, using 32 threads for the two cited steps, respectively.

Finally, we applied this new implementation to a series of heavy (Rn) and super-heavy elements (Cn, Fl, Og) embedded in a C_60_ cage to study the confinement effect induced by C_60_ on their electronic structure. An analysis of the embedding potential demonstrated that it can be well-approximated by a simple radial potential which is marginally affected by the nature of the central atom. These latter results let us clearly envision a practical approach to be used to build model potential as a result of the FDE procedure.

The current implementation is limited to the use of the DKS theory in the active subsystem embedded into a frozen environment without taking into account the mutual polarization effects between subsystems. However, the algorithms implemented here represent a solid starting point for future developments, including a DKS-in-DFT scheme in which one relaxes the electron density of one subsystem at a time keeping frozen the others, until electron densities of all subsystems reach a required convergence (freeze-and-thaw cycles) or subsystem real time DKS-in-DKS by evolving the subsystems in time simultaneously, while updating the embedding potential between the systems at every time step, extending the approach implemented in nonrelativistic context by Pavanello et al.^52^ The latter procedure would represent a significant advance for studying the energy transfer phenomena in molecular systems with strong spin–orbit coupling both in linear and nonlinear regimes.

## Supporting Information Available

Technical details related to a detailed description of the Python code; molecular geometries, dipole moment, and dipole polarizability for the water–ammonia adduct using water as an active system; description of the procedure to generate the density fitting basis set, G-spinor basis and fitting set used (for Rn, Cn, Fl, and Og); shift of orbital energies, with respect to the isolated Rn atom for the Rn–C60 system, using FDE scheme using Scalar ZORA with the SAPA (the Sum of neutral Atomical Potential Approximation), the MAPA (the Minuimum of neutral Atomical Potential Approximation) potentials and also introducing the mutual relaxation between subsystems via the “freeze-and-thaw cycles” scheme (freeze-and-thaw); evaluation of the spherical average of the EMBP; the full list of eigenvalues for neutral endohedral fullerenes (PDF)
