# Supplementary material for: Frozen-Density Embedding for Including Environmental Effects in the Dirac-Kohn–Sham Theory: An Implementation Based on Density Fitting and Prototyping Techniques
Source: J Chem Theory Comput. 2022 Sep 29;18(10):5992–6009. doi: 10.1021/acs.jctc.2c00499 (PMC9558305; doi:10.1021/acs.jctc.2c00499)
Supplement: Supplementary file 1 — ct2c00499_si_001.pdf [file ct2c00499_si_001.pdf]

Supporting Information: Frozen-Density Embedding for including environmental effects in the Dirac-Kohn-Sham theory: an implementation based on density fitting and prototyping techniques

Matteo De Santis,[†] Diego Sorbelli,[‡,¶] Valérie Vallet,[†] André Severo Pereira Gomes,[†] Loriano Storchi,[*,§,¶] and Leonardo Belpassi[*,¶]

†*Univ. Lille, CNRS, UMR 8523-PhLAM-Physique des Lasers Atomes et Molécules, F-59000 Lille, France*

‡*Dipartimento di Chimica, Biologia e Biotecnologie, Università degli Studi di Perugia, Via Elce di Sotto 8, 06123 Perugia, Italy*

¶*Istituto di Scienze e Tecnologie Chimiche (SCITEC), Consiglio Nazionale delle Ricerche c/o Dipartimento di Chimica, Biologia e Biotecnologie, Università degli Studi di Perugia, Via Elce di Sotto 8, 06123 Perugia, Italy*

§*Dipartimento di Farmacia, Università degli Studi 'G. D'Annunzio', Via dei Vestini 31, 66100 Chieti, Italy*

E-mail: loriano@storchi.org; leonardo.belpassi@cnr.it

1 Description of the Python code: PyBERTHAembed

Herein, we present a detailed description of the newly developed **pyberthaemb.py** code. It is composed of two main modules: the **pyembmod** one, that allows to manage all the important quantities for the FDE implementation, and the **pyberthamod** module.[S1]

Specifically, the **pyemb** class inside the **pyembmod** module allows to well isolate all the FDE data and operations increasing the level of abstraction. The module is used to manage all the required quantities for the generation of the embedding potential, that is $v_{\mathrm{emb}}[\tilde{\rho}_I, \rho_{II}(\boldsymbol{r})]$. It has been engineered in a such manner that all details of the FDE low-lying implementation will be completely transparent from the PyBERTHA side. This has the advantage that all future developments and/or integration of the FDE scheme (g.e. using DKS theory also for the environment DKS-in-DKS FDE) will not affect the PyBERTHAembed code, i.e., it will remain completely unchanged. In particular in this first version, the **pyembmod** module can handle the basic procedures previously implemented in the Psi4-rt-PyEmbed software which are based on the use of PyADF,[S2,S3] PyEmbed module,[S4,S5] and the XCFun library[S6,S7] to evaluate non-additive exchange-correlation and kinetic energy contributions on a user-defined integration grids. This approach gave us both the advantages of the code re-usability and, even more importantly, a DFT-in-DFT FDE reference implementation in which we can have the precise control over all those details and parameters from which a FDE scheme depends on (i.e., algorithms, numerical grid definition, quantum chemistry packages used to determine electronic density and Coulomb potential of the environment, basis sets, exchange-correlation functionals, etc.). This has clearly made the debugging phase in the development of PyBERTHAembed software straightforward.

Algorithm 1 reports the most important part of the **pyberthaemb.py** code, and it well illustrates how we can gain a relatively simple workflow to implement FDE using the DKS level of theory for the active system using PyBERTHA and the new **pyemb** class for the environmental system. The **pybertha** class is instantiated (line 4) with the shared object **bertha_wrapper.so** specified as an input. The SO contains the cited **c_wrapper** and

Algorithm 1 Illustrative Python code to compute active system DKS density (using the **pyberthaemb.py** code), environment density and Coulomb potential (using the ADF code) and non-additive embedding potential via the **pyemb** module

```
import berthamod
import pyembmod
...
bertha = berthamod.pybertha(pberthaopt.wrapperso)
# set options for the DKS calculation
bertha.set_fnameinput(fnameinput)
bertha.set_fittfname(fittfname)
...
bertha.init()
ovapm, eigem, fockm, eigen = bertha.run()
...
activefname = pberthaopt.activefile
envirofname = pberthaopt.envirofile
embfactory = pyembmod.pyemb(activefname,envirofname,'adf') #job-type='adf' is default
embfactory.set_options(param=pberthaopt.param,....) # several paramenters to be specified in input- e.g AUG/ADZP for ADF
# embfactory.set_grid_filename(pberthaopt.gridfname) # a general grid may be used
...
embfactory.initialize()
grid = embfactory.get_grid()
...
rho = bertha.get_density_on_grid(grid)
density=numpy.zeros((rho.shape[0],10))
density[:,0] = rho
...
pot = embfactory.get_potential(density)
...
for out_iter in range (maxiter): # split-SCF scheme iterations, see text
 bertha.init()
 ...
 bertha.set_embpot_on_grid(grid, pot)
 ovapm, eigem, fockm, eigen = bertha.run(eigem)
 rho = bertha.get_density_on_grid(grid)
 density=numpy.zeros((rho.shape[0],10))
 density[:,0] = rho
 pot_old=pot
 pot = embfactory.get_potential(density)
 norm_pot = numpy.sqrt(numpy.sum((pot-pot_old)**2))
 norm_D = numpy.linalg.norm(diffD,'fro')
 norm_D = numpy.linalg.norm(diffD,'fro')
 if (norm_D<(1.0e-6) and norm_pot <(1.0e-4)):
 bertha.finalize()
 break
```

bertha_wrapper code which are based on the core FORTRAN libraries, namely: **libbertha.so** and **libberthaserial.so** (see Figure 1 in the main text). After the initialization, the full DKS calculation is worked out (line 10) using the **bertha.run()** method. At line 14 the **pyemb** class is also instantiated specifying the files (specified in xyz format) for the geometries of both the active and embedding systems. The quantum chemistry software employed for the actual calculation of the environment system is specified at this stage (in the current example, and throughout this work, we used the ADF package[S8]).

All the details for the computation of the embedding system are set at line 15. This includes: the selection of the type of basis set functions, Hamiltonian, exchange-correlation functional and also the non-additive kinetic functional used to define the embedding potential. At this stage all the basis sets and exchange-correlation functionals available in the ADF library can be used. Similarly, the numerical integration grid used for the numerical representation of the embedding potential is set, both the type (global grid or active system) and the quality. By default the numerical grids internally defined by the ADF program are used, however other options are available, including the possibility to use an user-defined grid (see for instance line 16, commented).

The **embfactory.initialize()** method performs a stand-alone single point calculation on the embedding system. The method evaluates the nuclear and Coulomb potentials of the environment and its ground state electron density (ρ_{II}). All these quantities are mapped on the numerical grid. At this time, the numerical grid defined within PyADF is made available (as a **numpy.array**) using the **get_grid()** method (line 19) and used as an input for the **get_density_on_grid()** method of the **pybertha** class. This method allows to define the ground state density $\tilde{\rho}_I$ of the active system at DKS level of theory. $\tilde{\rho}_I$ is also available as a **numpy.array** that, after a reshape (line 22), can be used as an input of the **get_potential()** method of the **pyemb** class (line 25) to obtain the final embedding potential.

After this initial setup, we proceed to the actual FDE calculation. In this example, the

embedding potential will be generated using the active subsystem density (loop structure, lines 27 to 40), using the split-SCF scheme.[S9] Thus, in each of the spin-SCF iterations, the new **set_embpot_on_grid()** method of **pybertha** class makes both the numerical grid and the embedding potential available at the FORTRAN layer. Thus, the numerical integration of the $v^{emb}(\mathbf{r})$ on the fitting basis functions (Eq.32 in the main text) and linear system solution (Eq. 31 in the main text) are efficiently evaluated in FORTRAN. The DKS matrix employed in the intervening BERTHA calculation (**bertha.run()**, line 31) is updated using the G-spinor representation of the embedding potential, and here the split-SCF scheme is interesting as it does not require the evaluation of the embedding potential at each SCF step taking place on the BERTHA side. The new fitted density (line 32) is used to compute a new embedding potential (line 36) which is used in for the next iteration of the split-SCF procedure.[S9] This scheme is iterated till a convergence criteria is satisfied (line 40).

2 Initial validation in comparison with Psi4-rt-PyEmbed code: H_2O-NH_3

Table S1: Geometry (Å) of the adduct where water molecule is the active system that is bound to an ammonia molecule, which instead plays the role of the embedding environment.

H_2O			
Atom	X	Y	Z
O	1.568501	0.105892	0.000005
H	0.606736	-0.033962	-0.000628
H	1.940519	-0.780005	0.000222
NH_3			
Atom	X	Y	Z
N	-1.395591	-0.021564	0.000037
H	-1.629811	0.961096	-0.106224
H	-1.862767	-0.512544	-0.755974
H	-1.833547	-0.330770	0.862307

In all the calculations the water molecule is the active system that is bound to an ammonia molecule, which instead plays the role of the embedding environment. In the Psi4-rt-PyEmbed case we use basis sets obtained by the decontraction of the Gaussian cc-pVDZ, cc-pVTZ and aug-cc-pVDZ basis sets[S10,S11] for the active system (these basis sets are referred as cc-pvdz-decon, cc-pvtz-decon and aug-cc-pvdz-decon, respectively). The same basis set has been used in the DKS calculation to define the large component of the G-spinor basis set. The corresponding small component was generated using restricted kinetic balance relation.[S12] Noteworthy, for these reference calculations, we have used an extremely large auxiliary fitting basis set ($A4_{spdfg}$) which gives an error on the Coulomb energy even below 10^{-6} Eh. The computational details for the definition of the environment, including parameters to define the embedding potential, are identical in both PyBERTHAembed and Psi4-rt-PyEmbed. In particular, the basis set used in PyADF for the calculation of the environment frozen density (ammonia) and the embedding potential is the AUG-TZ2P Slater-type set from the ADF library.[S13] As numerical grid we used the supermolecular Voronoi Polyhedra grid defined in ADF which is set defining an the integration parameter equal to 4 (this corresponds

to a total number of 33280 grid points). The PBE[S14] exchange-correlation functional has been used for the active system while the BLYP[S15,S16] exchange-correlation functional has been used for the ammonia molecule. The Thomas-Fermi and LDA functionals[S17,S18] has been employed for the non-additive kinetic and non-additive exchange-correlation potential, respectively. The effect of the environment (ammonia) on the active system (water) have been evaluated comparing the dipole moment components and diagonal elements of the polarizability tensor (α_{xx}, α_{yy} and α_{zz}) of the isolated (Free) respect to the embedded (Emb) water. We note here that the quoted polarizability values are not those for the supermolecular system, but only for the active subsystem.

The numerical results, reported in Table S3, show an evident quantitative agreement between the two implementations. Indeed, both the variations induced by the presence of the embedding system (Δ values) and the absolute values show a good agreement. Noteworthy, independently by the basis set used, the differences are below of 0.001 a.u. and 0.01 a.u. for the dipole moment components and for the polarizability tensor components, respectively. We mention that we also performed the calculations increasing the speed of light by 1 order of magnitude (i.e., c = 1370.36 a.u.) to approximate the non-relativistic limit and, as expected, we obtain almost indistinguishable results (see Table S.3). All the above findings make us confident that our implementation is both numerically stable and correct.

Table S2: Dipole moment (components μ_x, μ_y, μ_z and module $|\mu|$) and dipole polarizability (tensor diagonal components α_{xx}, α_{yy}, α_{zz} and isotropic contribution α_{iso}) of both the isolated (Free) and embedded (Emb) water molecule. In the embedded water molecule, an ammonia molecule is used as environment. Data have been obtained using our new PyBERTHAembed implementation and the reference Psi4-rt-PyEmbed implementation (see text for details). The shift Δ is also reported. All numerical data are reported in atomic units (a.u.). The diagonal components of the dipole polarizability tensor have been calculated using a finite field approach using an external electric of 0.001 a.u.

	Psi4-rt-PyEmbed			PyBERTHAembed		
	Free	Emb	Δ	Free	Emb	Δ
	a) aug-cc-pvdz-decon					
μ_x	-0.35403	-0.49351	-0.13948	-0.35328	-0.49279	-0.13951
μ_y	-0.62058	-0.62976	-0.00918	-0.61908	-0.62812	-0.00904
μ_z	-0.00025	-0.00026	-0.00001	-0.00025	-0.00027	-0.00002
$\|\mu\|$	0.71446	0.80009	0.08563	0.71279	0.79836	0.08557
α_{xx}	10.34	9.79	-0.55	10.36	9.80	-0.56
α_{yy}	9.91	10.26	0.35	9.92	10.27	0.35
α_{zz}	9.62	10.16	0.54	9.64	10.18	0.54
α_{iso}	9.96	10.07	0.11	9.97	10.08	0.11
	b) cc-pvdz-decon					
μ_x	-0.38085	-0.50083	-0.11998	-0.37992	-0.50006	-0.12014
μ_y	-0.67072	-0.67187	-0.00115	-0.66911	-0.67023	-0.00112
μ_z	-0.00027	-0.00031	-0.00004	-0.00027	-0.00031	-0.00004
$\|\mu\|$	0.77130	0.83800	0.06670	0.76944	0.83622	0.06678
α_{xx}	7.35	6.79	-0.56	7.36	6.80	-0.56
α_{yy}	6.27	6.38	0.11	6.28	6.39	0.11
α_{zz}	3.70	3.71	0.01	3.70	3.71	0.01
α_{iso}	5.77	5.63	-0.14	5.78	5.63	-0.15
	c) cc-pvtz-decon					
μ_x	-0.36464	-0.49344	-0.12880	-0.36377	-0.49267	-0.12890
μ_y	-0.64037	-0.64583	-0.00546	-0.63883	-0.64430	-0.00547
μ_z	-0.00025	-0.00028	-0.00003	-0.00026	-0.00028	-0.00002
$\|\mu\|$	0.73691	0.81276	0.07585	0.73514	0.81108	0.07593
α_{xx}	8.52	7.95	-0.57	8.53	7.96	-0.57
α_{yy}	7.80	7.94	0.14	7.81	7.95	0.14
α_{zz}	5.77	5.80	0.03	5.78	5.82	0.04
α_{iso}	7.36	7.23	-0.13	7.37	7.24	-0.13

Table S3: Dipole moment (components μ_x, μ_y, μ_z and module $|\mu|$) and dipole polarizability (tensor diagonal components α_{xx}, α_{yy}, α_{zz} and isotropic contribution α_{iso}) of both the isolated (free) and embedded (emb.) water molecule. In the embedded water molecule, an ammonia molecule is used as environment. Data have been obtained using our new pyberthaemb implementation increasing the speed of light c = 10 · 137.036 au.

	a) aug-cc-pvdz-decon		
	Free	Emb	Δ
μ_x	-0.35424	-0.49352	-0.13928
μ_y	-0.62075	-0.62973	-0.00898
μ_z	-0.00024	-0.00027	-0.00003
$\|\mu\|$	0.71471	0.80007	0.08536
α_{xx}	10.35	9.79	-0.56
α_{yy}	9.91	10.26	0.35
α_{zz}	9.62	10.16	0.54
α_{iso}	9.96	10.07	0.11

3 Fitting basis set

The exponents generation depends on the smallest and largest exponent in the primitive Gaussian exponents of the chosen basis set and by a parameter (N=2,3,4) which together determine the total number of exponents. We recall that the auxiliary functions we employed are grouped in s, sp, spd, spdf and spdfg sets and that the exponents are shared within each of these sets. For instance, the A4$_{spdfg}$ (generated with N=4) and associated with cc-pvtz basis for the oxygen atom have 17 different exponents with the angular part described with (5,7,5) auxiliary function notation adopted by Calaminici et al.[S19] It describes five s sets together with 5 functions, seven spd sets together with 70 functions, and 5 spdfg sets together with functions 175. For the hydrogen atom the A4$_{spdfg}$ fitting basis set correspond to a (2,4,3) fitting basis set. The total number of auxiliary function for the water molecule is 544. Using the same automatic generation scheme (even if may be not optimal) we generate different sets of exponents of reduced size depending on the teger parameter N. Thus, for the same cc-pvdz basis, we generate other five fitting basis sets (A2$_s$, A2$_{sp}$, A2$_{spd}$, A2$_{spdfg}$ and A3$_{spdfg}$). See the definition in the following.

```
Fitting basis set: A2s

H
5
13.14560 0
16.43200 0
3.28640  0
0.82160  0
0.20540  0

O
9
7012.35200 0
8765.44000 1
1753.08800 1
438.27200  1
109.56800  1
27.39200   1
6.84800    1
1.71200    1
0.42800    1

Fitting basis set: A2sp

H
5
13.14560 0
```

```
16.43200 1
3.28640  1
0.82160  1
0.20540  1

O
9
7012.35200 0
8765.44000 1
1753.08800 1
438.27200  1
109.56800  1
27.39200   1
6.84800    1
1.71200    1
0.42800    1
```

Fitting basis set: A2spd

```
H
5
13.14560 0
16.43200 2
3.28640  2
0.82160  2
0.20540  2
```

```
O
7012.35200 0
8765.44000 2
1753.08800 2
438.27200  2
109.56800  2
27.39200   2
6.84800    2
1.71200    2
0.42800    2
```

Fitting basis set: A2spdfg

```
H
5
13.14560 0
16.43200 2
3.28640  2
0.82160  4
0.20540  4

O
9
7012.35200 0
8765.44000 2
1753.08800 2
438.27200  2
```

```
109.56800  2
27.39200   2
6.84800    4
1.71200    4
0.42800    4
```

Fitting basis set: A3spdfg

```
H
7
14.45364 0
21.68046 2
4.81788  2
1.60596  2
0.53532  4
0.17844  4
0.05948  4
```

```
O
11
10837.45980 0
16256.18970 2
3612.48660  2
1204.16220  2
401.38740   2
133.79580   2
44.59860    2
```

```
14.86620    4
4.95540     4
1.65180     4
0.55060     4
```

Fitting basis set: A4spdfg

```
H
8
15.61600 0
31.23200 2
7.80800  2
3.90400  2
1.95200  2
0.97600  4
0.48800  4
0.24400  4
```

```
O
17
14024.70400 0
28049.40800 2
7012.35200  2
3506.17600  2
1753.08800  2
876.54400   2
438.27200   2
```

219.13600	2
109.56800	2
54.78400	2
27.39200	2
13.69600	4
6.84800	4
3.42400	4
1.71200	4
0.85600	4
0.42800	4

4 Computational time

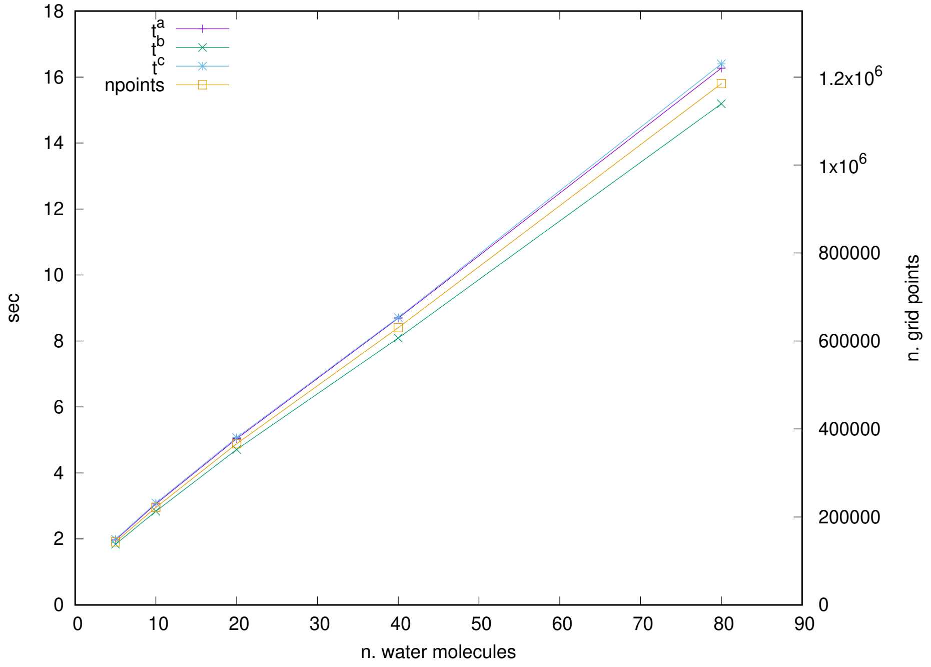

Figure S1: Computational time of the tasks associated with the FDE procedure (a,b, and c; see main text for the definitions) and the total number of grid points are reported for $Au_4@(H_2O)_n$ (with n equal to 5, 10, 20, 40 and 80). Time is in sec.

5 Fitting Basis set definition for Rn, Cn, Fl and Og

```
Rn
 25
 11.3695320E+07 0
 2.9358812E+07  0
 9.4982512E+06  0
 3.3357532E+06  0
 12.5034106E+05 2
 4.8936286E+05  2
 19.9232080E+04 2
 8.3919070E+04  2
 3.6483407E+04  2
 16.3264454E+03 2
 7.5044994E+03  2
 3.5359806E+03  2
 17.0195914E+02 2
 8.0823622E+02  2
 4.0781424E+02  2
 2.1070864E+02  2
 10.3445490E+01 2
 5.4675064E+01  2
 2.5256416E+01  2
 13.2706936E+00 2
 5.1310780E+00  4
 2.5895016E+00  4
 7.4055918E-01  4
 2.8874634E-01  4
```

8.8783890E-02 4

Cn

26

10.5525682E+07 0

2.8026742E+07 0

9.5036698E+06 0

3.5503500E+06 0

14.4081728E+05 2

6.1222764E+05 2

2.7032544E+05 2

12.2344004E+04 2

5.6558338E+04 2

2.6594564E+04 2

12.7103702E+03 2

6.1719122E+03 2

3.0521732E+03 2

15.2210270E+02 2

7.8252222E+02 2

4.1243852E+02 2

2.1889478E+02 2

11.9177684E+01 2

5.9408372E+01 2

3.3631760E+01 2

16.1254664E+00 2

8.7343090E+00 2

3.4773516E+00 4

16.6994090E-01 4

4.5980612E-01 4

16.4591954E-02 4

Fl

27

10.5100528E+07 0

2.7919038E+07 0

9.4708040E+06 0

3.5413462E+06 0

14.4108562E+05 2

6.1494150E+05 2

2.7307174E+05 2

12.4347488E+04 2

5.7844092E+04 2

2.7353736E+04 2

13.1373470E+03 2

6.4051104E+03 2

3.1786378E+03 2

15.9218248E+02 2

8.2096760E+02 2

4.3381696E+02 2

2.3128896E+02 2

12.6241812E+01 2

6.3432228E+01 2

3.6004852E+01 2

17.4614446E+00 2

9.5272666E+00 2

3.8762812E+00 4

19.0933950E-01 4

5.7274828E-01 4

2.1504274E-01 4

4.4258228E-02 4

Og

27

10.4908686E+07 0

2.7868550E+07 0

9.4517386E+06 0

3.5331196E+06 0

14.4000862E+05 2

6.1668480E+05 2

2.7560868E+05 2

12.651001E+04 2

5.9375108E+04 2

2.8313948E+04 2

13.6997362E+03 2

6.7204318E+03 2

3.3536848E+03 2

16.9430534E+02 2

8.7817464E+02 2

4.6504244E+02 2

2.5194358E+02 2

13.8681056E+01 2

```
7.0841358E+01  2
4.0483622E+01  2
2.0105508E+01  2
11.1240528E+00 2
4.7031744E+00  4
2.4220012E+00  4
8.2445578E-01  4
3.1806344E-01  4
8.7080594E-02  4
```

6 Evaluation of the spherical average of the EMBP

As in the case of the the contour plot, reported in the main text, it is important to underlined as the spherical average of the EMBP for the neutral endohedral fullerenes A@C_{60} (A=Rn,Og,Fl,Cn) is the result of a Nearest-neighbor interpolation performed starting from the potential represented on the original non homogeneous ADF grid. Initially we took advantage of the capability of PyADF to dump the Embedding Potential into a file. Subsequently the file is processed via a simple Python script (i.e. **gridtodx.py**[S20]) that, using a Nearest neighbor interpolation, as implemented in the SciPy,[S21] transforms the potential, originally represented on a non homogeneous grid to a potential evaluated on a homogeneous grid, producing a DX file via the gridDataFormats package.[S22] Once the EMBP has been dumped in a DX file, using an homogeneously spaced grid, quite easily the Spherical overage can be evaluated (i.e. **avgalongr.py**[S20]) simply considering the average of the Embedding Potential values contained in spherical shells, always centered in the A atom, of increasing radius and constant thickness dr.

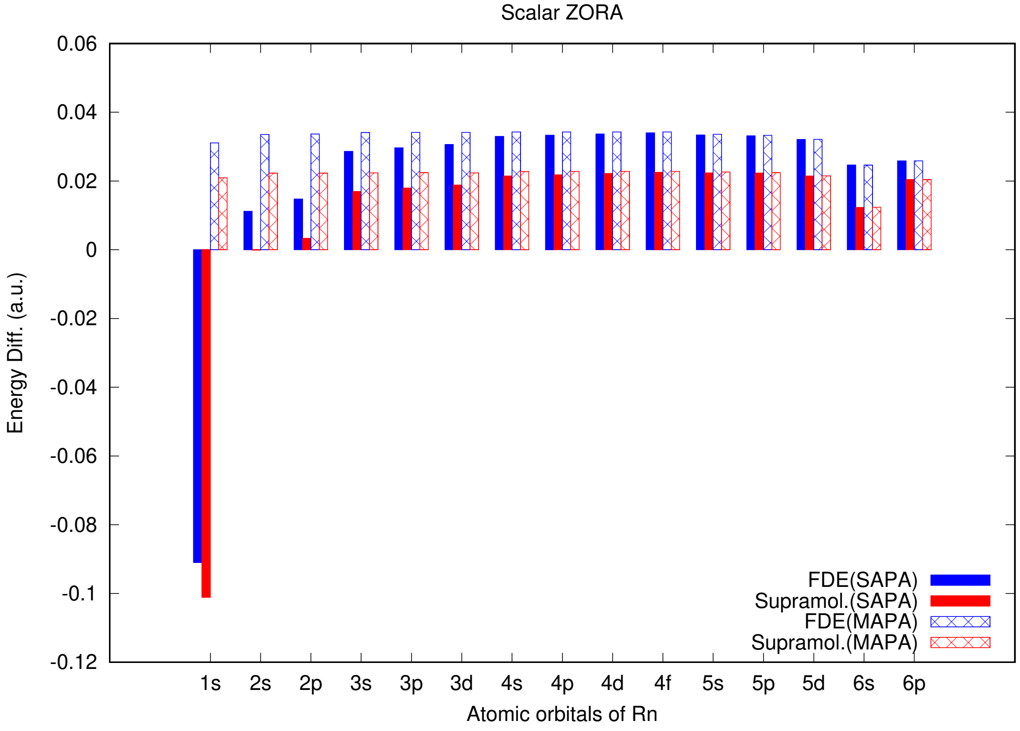

Figure S2: Differences in orbital energies with respect to the isolated Rn atom for the Rn-C_{60} system using the frozen density embedding (FDE) scheme (where Rn is used as active system while C_{60} as frozen environment) and the supramolecular calculation (Supramol.). All calculations have been carried out with the ADF code (2019.307 version)[S8] using the BLYP functional together with the TZP basis set, the ZORA Hamiltonian for the inclusion of scalar relativistic effects, no frozen core approximation and the quality of both the density fitting and the numerical integration set to "Very good". The FDE calculations have been carried out using a full supramolecular grid for the frozen subsystem. The results are reported for two different methods used to implement the ZORA Hamiltonian in ADF: the SAPA (the Sum of neutral Atomical potential Approximation) and the MAPA (the Minumium of neutral Atomical potential Approximation). The former has been the default in ADF till the version ADF2016. See ADF manual for details (https://www.scm.com/doc.2019/ADF/_downloads/ADF.pdf, pag.73).

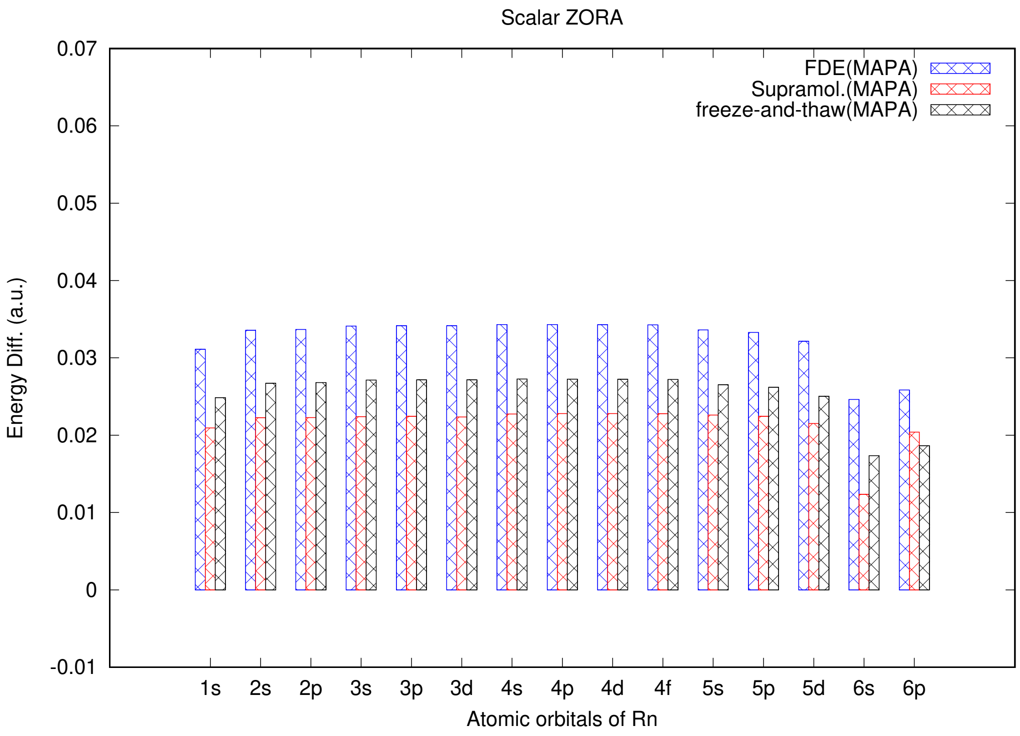

Figure S3: Differences in orbital energies with respect to the isolated Rn atom for the Rn-C_{60} system using the frozen density embedding (FDE) scheme (where Rn is used as active system while C_{60} as frozen environment) and introducing the mutual relaxation between subsystems via the "freeze-and-thaw cycles" scheme (freeze-and-thaw). The results for the supramolecular calculation (Supramol.) are also reported. All calculations have been carried out with the ADF code (2019.307 version)[S8] using the BLYP functional together with the TZP basis set, the ZORA Hamiltonian for the inclusion of scalar relativistic effects, no frozen core approximation and the quality of both the density fitting and the numerical integration set to "Very good". The FDE calculations have been carried out using a full supramolecular grid for the frozen subsystem. The results are reported using scalar ZORA Hamiltonian using the MAPA (Minumium of neutral Atomical potential Approximation) method.

Table S4: Atomic Orbital energy of Rn (1s) embedded in C_{60}, energy shift respect to the isolated Rn atom and HOMO-LUMO gap. Data are obtained using different model potentials (see main text for the definitions). A graphical picture of the Multi Steps Model Potential is given in Figure S4.

	1s orbital energy (a.u.)	1s shift respect to isolated atom	HOMO-LUMO gap (a.u.)
SMP	-3614.919179	-0.030386	0.118722
FDE	-3614.882044	0.006749	0.209643
EMBP Spherical Average	-3614.882268	0.006525	0.207990
Multi Steps Model Potential	-3614.878363	0.011317	0.203040

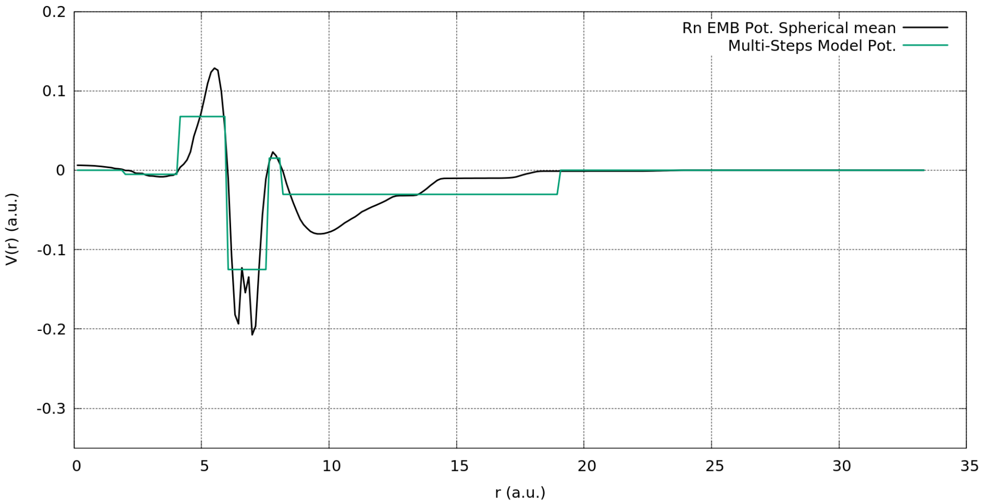

Figure S4: Multi Steps Model Potentials. EMBP Spherical Average has been also reported for easy reference.

7 Eigenvalues for neutral endohedral fullerenes A@C_{60} (A=Rn,Og,Fl,Cn)

Table S5: Rn atom eigenvalues calculations were carried out using a basis set for the Rn atom generated by uncontracting triple–ζ quality Dyall's basis sets[S23–S26] augmented with the related polarization and correlating functions. Final basis set schemes is (31s27p18d12f4g1h). For all the elements we used auxiliary basis sets already employed in Ref. S27 and are explicitly reported in Section 5. The eigenvalues are degenerate in pairs (Kramer-pairs) and, for the reader convenience, we report only the eigenvalues corresponding to the eigenvectors labeled with odd number.

Eigenvalue	Isolated (a.u.)	SPM (a.u.)	FDE C_{60} (a.u.)	EMBP Spherical Average (a.u.)
1	-3614.89008690	-3614.91899840	-3614.88204411	-3614.88244268
3	-657.66880806	-657.69759019	-657.66072974	-657.66108023
5	-633.13630845	-633.16511925	-633.12823503	-633.12859842
7	-532.03165711	-532.06044938	-532.02357799	-532.02393103
9	-532.03165711	-532.06044925	-532.02357798	-532.02393093
11	-161.24972086	-161.27857149	-161.24161423	-161.24186586
13	-150.03564968	-150.06449386	-150.02754166	-150.02780118
15	-127.16985627	-127.19871977	-127.16175337	-127.16199774
17	-127.16985627	-127.19871943	-127.16175334	-127.16199754
19	-108.82681068	-108.85563632	-108.81870354	-108.81896751
21	-108.82681068	-108.85563594	-108.81870350	-108.81896734
23	-104.03878498	-104.06761653	-104.03067811	-104.03093824
25	-104.03878498	-104.06761619	-104.03067810	-104.03093681
27	-104.03878498	-104.06761482	-104.03067805	-104.03093639
29	-38.41192817	-38.44100309	-38.40395119	-38.40402526
31	-33.45435309	-33.48342696	-33.44637606	-33.44645131
33	-27.70583185	-27.73492964	-27.69787781	-27.69792827
35	-27.70583185	-27.73492935	-27.69787775	-27.69792780
37	-19.64898911	-19.67807877	-19.64103414	-19.64108703
39	-19.64898911	-19.67807796	-19.64103406	-19.64108669

41	-18.57803597	-18.60713766	-18.57008767	-18.57014407
43	-18.57803597	-18.60713371	-18.57008755	-18.57012884
45	-18.57803597	-18.60712103	-18.57008722	-18.57012625
47	-8.06011789	-8.08919929	-8.05215929	-8.05222090
49	-8.06011789	-8.08919622	-8.05215921	-8.05220771
51	-8.06011789	-8.08918498	-8.05215890	-8.05220556
53	-7.80133550	-7.83042125	-7.79338052	-7.79344061
55	-7.80133550	-7.83041899	-7.79338046	-7.79342977
57	-7.80133550	-7.83041078	-7.79338019	-7.79342535
59	-7.80133550	-7.83040393	-7.79338008	-7.79341997
61	-7.36183057	-7.39082933	-7.35407337	-7.35398408
63	-5.56245790	-5.59142512	-5.55473911	-5.55464706
65	-4.35897913	-4.38787434	-4.35134711	-4.35125165
67	-4.35897913	-4.38787319	-4.35134692	-4.35125060
69	-1.77712390	-1.80575462	-1.76981056	-1.76972637
71	-1.77712390	-1.80575251	-1.76981027	-1.76972561
73	-1.61468572	-1.64324492	-1.60745552	-1.60737416
75	-1.61468572	-1.64324255	-1.60745537	-1.60737269
77	-1.61468572	-1.64324207	-1.60745512	-1.60737182
79	-0.79171100	-0.81701374	-0.78769031	-0.78760564
81	-0.37400268	-0.39744765	-0.37107930	-0.37098221
83	-0.24311329	-0.26707774	-0.24019052	-0.24012615
85	-0.24311329	-0.26706790	-0.24018861	-0.24012134
lumo	-0.02215500	-0.14838813	-0.03054620	-0.03213182

Table S6: Cn atom eigenvalues calculations were carried out using a basis set for the Rn atom generated by uncontracting triple–ζ quality Dyall's basis sets[S23–S26] augmented with the related polarization and correlating functions. Final basis set schemes is (32s29p20d14f7g2h). For all the elements we used auxiliary basis sets already employed in Ref. S27 and are explicitly reported in Section 5. The eigenvalues are degenerate in pairs (Kramer-pairs) and, for the reader convenience, we report only the eigenvalues corresponding to the eigenvectors labeled with odd number.

Eigenvalue	Isolated (a.u.)	SPM (a.u.)	FDE C_{60} (a.u.)	EMBP Spherical Average (a.u.)
1	-7032.45007220	-7032.44923436	-7032.44464253	-7032.44500826
3	-1428.41195749	-1428.41156637	-1428.40670958	-1428.40705819
5	-1393.08239459	-1393.08185954	-1393.07710728	-1393.07746094
7	-994.59221688	-994.59189562	-994.58698971	-994.58733567
9	-994.59221688	-994.59189560	-994.58698970	-994.58733567
11	-382.30405390	-382.30482963	-382.29905954	-382.29937216
13	-364.64343793	-364.64407005	-364.63840800	-364.63872435
15	-268.36169388	-268.36274504	-268.35677565	-268.35708207
17	-268.36169388	-268.36274493	-268.35677564	-268.35708203
19	-240.28577774	-240.28650376	-240.28077882	-240.28109278
21	-240.28577774	-240.28650365	-240.28077881	-240.28109275
23	-223.83085096	-223.83169143	-223.82587977	-223.82619077
25	-223.83085096	-223.83169134	-223.82587977	-223.82619063
27	-223.83085096	-223.83169081	-223.82587976	-223.82619060
29	-108.88021773	-108.88292836	-108.87578446	-108.87604491
31	-100.16924690	-100.17188767	-100.16479372	-100.16505663
33	-72.76445385	-72.76761411	-72.76015200	-72.76040646
35	-72.76445385	-72.76761406	-72.76015196	-72.76040643
37	-59.12332404	-59.12636949	-59.11899023	-59.11924693
39	-59.12332404	-59.12636938	-59.11899019	-59.11924687

41	-54.69677467	-54.69995406	-54.69247766	-54.69273389
43	-54.69677467	-54.69995221	-54.69247762	-54.69273153
45	-54.69677467	-54.69994237	-54.69247757	-54.69273082
47	-36.79274450	-36.79567771	-36.78836720	-36.78862774
49	-36.79274450	-36.79567634	-36.78836717	-36.78862585
51	-36.79274450	-36.79566842	-36.78836712	-36.78862529
53	-35.49140797	-35.49440957	-35.48705004	-35.48730947
55	-35.49140797	-35.49440657	-35.48705002	-35.48730800
57	-35.49140797	-35.49440309	-35.48705000	-35.48730685
59	-35.49140797	-35.49439666	-35.48704995	-35.48730625
61	-28.02372229	-28.02842357	-28.01984552	-28.02004574
63	-24.11984009	-24.12457622	-24.11597417	-24.11617201
65	-16.28263942	-16.28787031	-16.27892117	-16.27908354
67	-16.28263942	-16.28786916	-16.27892106	-16.27908310
69	-10.57527237	-10.58063727	-10.57160066	-10.57175746
71	-10.57527237	-10.58063652	-10.57160050	-10.57175714
73	-9.46617915	-9.47168146	-9.46254743	-9.46270395
75	-9.46617915	-9.47167368	-9.46254728	-9.46269557
77	-9.46617915	-9.47163824	-9.46254713	-9.46269273
79	-4.93682232	-4.94335514	-4.93360189	-4.93374040
81	-3.53509878	-3.54172862	-3.53195095	-3.53209276
83	-2.75156393	-2.75740433	-2.74806229	-2.74821074
85	-2.75156393	-2.75739827	-2.74806212	-2.74820447
87	-2.75156393	-2.75737121	-2.74806191	-2.74820214
89	-2.51939601	-2.52529935	-2.51591765	-2.51606512
91	-2.51939601	-2.52528945	-2.51591751	-2.51606046
93	-2.51939601	-2.52527896	-2.51591741	-2.51605694

95	-2.51939601	-2.52525820	-2.51591721	-2.51605501
97	-1.92297586	-1.92981522	-1.92019433	-1.92034235
99	-1.92297586	-1.92981308	-1.92019378	-1.92034154
101	-0.36730789	-0.37391233	-0.36577740	-0.36590764
103	-0.36730789	-0.37391079	-0.36577638	-0.36590700
105	-0.33865992	-0.34563112	-0.33893155	-0.33903818
107	-0.25067057	-0.25725550	-0.24947103	-0.24959746
109	-0.25067057	-0.25725075	-0.24947005	-0.24959717
111	-0.25067057	-0.25723853	-0.24946933	-0.24959560
lumo	-0.09869815	-0.20143106	-0.10478383	-0.10490259

Table S7: Fl atom eigenvalues calculations were carried out using a basis set for the Rn atom generated by uncontracting triple–ζ quality Dyall's basis sets[S23–S26] augmented with the related polarization and correlating functions. Final basis set schemes is (31s30p21d14f6g2h). For all the elements we used auxiliary basis sets already employed in Ref. S27 and are explicitly reported in Section 5. The eigenvalues are degenerate in pairs (Kramer-pairs) and, for the reader convenience, we report only the eigenvalues corresponding to the eigenvectors labeled with odd number.

Eigenvalue	Isolated (a.u.)	SPM (a.u.)	FDE C_{60} (a.u.)	EMBP Spherical Average (a.u.)
1	-7384.82059694	-7384.86441378	-7384.81812651	-7384.81782805
3	-1513.68301889	-1513.72596392	-1513.68057206	-1513.68027823
5	-1478.65448296	-1478.69757441	-1478.65204844	-1478.65175426
7	-1037.05154341	-1037.09440831	-1037.04908835	-1037.04879453
9	-1037.05154341	-1037.09440813	-1037.04908834	-1037.04879451
11	-407.16520100	-407.20797661	-407.16253493	-407.16223615
13	-389.21052846	-389.25331925	-389.20788586	-389.20758722
15	-282.12499267	-282.16775599	-282.12228668	-282.12198839
17	-282.12499267	-282.16775567	-282.12228666	-282.12198835

19	-253.21978789	-253.26251748	-253.21713673	-253.21683868
21	-253.21978789	-253.26251718	-253.21713671	-253.21683864
23	-235.36191460	-235.40464237	-235.35924348	-235.35894541
25	-235.36191460	-235.40464202	-235.35924347	-235.35894507
27	-235.36191460	-235.40464192	-235.35924346	-235.35894504
29	-117.25255536	-117.29531109	-117.24965499	-117.24935632
31	-108.27676416	-108.31952250	-108.27387308	-108.27357481
33	-77.59480300	-77.63755002	-77.59184571	-77.59155651
35	-77.59480300	-77.63754946	-77.59184568	-77.59155651
37	-63.48169916	-63.52443735	-63.47875824	-63.47846788
39	-63.48169916	-63.52443680	-63.47875822	-63.47846779
41	-58.62892086	-58.67165757	-58.62596274	-58.62567909
43	-58.62892086	-58.67165745	-58.62596268	-58.62567291
45	-58.62892086	-58.67165344	-58.62596264	-58.62567227
47	-40.13178199	-40.17451891	-40.12884579	-40.12855744
49	-40.13178199	-40.17451882	-40.12884574	-40.12855252
51	-40.13178199	-40.17451563	-40.12884570	-40.12855207
53	-38.69576358	-38.73850113	-38.69281910	-38.69253245
55	-38.69576358	-38.73850046	-38.69281904	-38.69252824
57	-38.69576358	-38.73849845	-38.69281902	-38.69252681
59	-38.69576358	-38.73849658	-38.69281899	-38.69252463
61	-30.89239614	-30.93511873	-30.88923014	-30.88895927
63	-26.81395743	-26.85667911	-26.81078844	-26.81051672
65	-17.91416501	-17.95686734	-17.91093432	-17.91064622
67	-17.91416501	-17.95686620	-17.91093427	-17.91064588
69	-11.93662921	-11.97930797	-11.93338985	-11.93309840
71	-11.93662921	-11.97930672	-11.93338983	-11.93309831

73	-10.69339104	-10.73606051	-10.69014066	-10.68985912
75	-10.69339104	-10.73605948	-10.69014041	-10.68983896
77	-10.69339104	-10.73604667	-10.69014034	-10.68983653
79	-5.80073609	-5.84328165	-5.79743355	-5.79713126
81	-4.28884501	-4.33140900	-4.28555277	-4.28525578
83	-3.60491676	-3.64754878	-3.60164741	-3.60136013
85	-3.60491676	-3.64754854	-3.60164720	-3.60134440
87	-3.60491676	-3.64753820	-3.60164718	-3.60134265
89	-3.33398980	-3.37661888	-3.33071714	-3.33043068
91	-3.33398980	-3.37661688	-3.33071701	-3.33041833
93	-3.33398980	-3.37661100	-3.33071689	-3.33041430
95	-3.33398980	-3.37660554	-3.33071686	-3.33040787
97	-2.35606244	-2.39862058	-2.35292338	-2.35264626
99	-2.35606244	-2.39861653	-2.35292331	-2.35264616
101	-0.61967527	-0.66114973	-0.61738671	-0.61711598
103	-0.61967527	-0.66114357	-0.61738646	-0.61711547
105	-0.47926140	-0.51603299	-0.47925086	-0.47898960
107	-0.46293263	-0.50379743	-0.46099381	-0.46073029
109	-0.46293263	-0.50378996	-0.46099352	-0.46072341
111	-0.46293263	-0.50378741	-0.46099333	-0.46072233
113	-0.17795820	-0.21854531	-0.17662058	-0.17646775
lumo	-0.05245587	-0.14629461	-0.06614950	-0.06600113

Table S8: Og atom eigenvalues calculations were carried out using a basis set for the Rn atom generated by uncontracting triple–ζ quality Dyall's basis sets[S23–S26] augmented with the related polarization and correlating functions. Final basis set schemes is (31s30p21d14f6g2h). For all the elements we used auxiliary basis sets already employed in Ref. S27 and are explicitly reported in Section 5. The eigenvalues are degenerate in pairs (Kramer-pairs) and, for the reader convenience, we report only the eigenvalues corresponding to the eigenvectors labeled with odd number.

Eigenvalue	Isolated (a.u.)	SPM (a.u.)	FDE C_{60} (a.u.)	EMBP Spherical Average (a.u.)
1	-8143.60941658	-8143.66233440	-8143.59921010	-8143.59871400
3	-1700.86804673	-1700.92078516	-1700.85776874	-1700.85727112
5	-1668.29159261	-1668.34442448	-1668.28132288	-1668.28082556
7	-1125.16260496	-1125.21532797	-1125.15230582	-1125.15180752
9	-1125.16260496	-1125.21532783	-1125.15230580	-1125.15180743
11	-461.86386763	-461.91643716	-461.85342109	-461.85291813
13	-443.71587653	-443.76846448	-443.70544946	-443.70494673
15	-311.15028068	-311.20283821	-311.13979392	-311.13929226
17	-311.15028068	-311.20283793	-311.13979389	-311.13929207
19	-280.55110644	-280.60366650	-280.54066275	-280.54016058
21	-280.55110644	-280.60366624	-280.54066273	-280.54016041
23	-259.61197021	-259.66452095	-259.60151002	-259.60100805
25	-259.61197021	-259.66452014	-259.60151001	-259.60100762
27	-259.61197021	-259.66451984	-259.60150998	-259.60100749
29	-135.92353208	-135.97601471	-135.91289556	-135.91239629
31	-126.51483911	-126.56732858	-126.50421076	-126.50371197
33	-88.06705420	-88.11950988	-88.05636705	-88.05587612
35	-88.06705420	-88.11950947	-88.05636698	-88.05587588
37	-72.99161955	-73.04407872	-72.98094594	-72.98045433
39	-72.99161955	-73.04407823	-72.98094589	-72.98045401

41	-67.19151145	-67.24397130	-67.18082370	-67.18033966
43	-67.19151145	-67.24395544	-67.18082361	-67.18033220
45	-67.19151145	-67.24395482	-67.18082354	-67.18033104
47	-47.49861849	-47.55108521	-47.48795031	-47.48746172
49	-47.49861849	-47.55107297	-47.48795024	-47.48745587
51	-47.49861849	-47.55107234	-47.48795018	-47.48745507
53	-45.76283759	-45.81530302	-45.75216247	-45.75167576
55	-45.76283759	-45.81529122	-45.75216243	-45.75167138
57	-45.76283759	-45.81529061	-45.75216237	-45.75166837
59	-45.76283759	-45.81528362	-45.75216231	-45.75166655
61	-37.53884950	-37.59116109	-37.52802571	-37.52756092
63	-33.13104298	-33.18335364	-33.12021840	-33.11975295
65	-21.67975586	-21.73199441	-21.66889172	-21.66841096
67	-21.67975586	-21.73199387	-21.66889155	-21.66841082
69	-15.14881823	-15.20103315	-15.13795297	-15.13746947
71	-15.14881823	-15.20103248	-15.13795284	-15.13746910
73	-13.60019245	-13.65241183	-13.58932298	-13.58884904
75	-13.60019245	-13.65236864	-13.58932272	-13.58882915
77	-13.60019245	-13.65236770	-13.58932255	-13.58882553
79	-8.01197874	-8.06381481	-8.00117137	-8.00066332
81	-6.27745122	-6.32923085	-6.26667305	-6.26616767
83	-5.76088057	-5.81301519	-5.75002052	-5.74953948
85	-5.76088057	-5.81298110	-5.75002032	-5.74952348
87	-5.76088057	-5.81297997	-5.75002017	-5.74952090
89	-5.40095224	-5.45307370	-5.39009381	-5.38961280
91	-5.40095224	-5.45304449	-5.39009373	-5.38960178
93	-5.40095224	-5.45304297	-5.39009357	-5.38959443

95	-5.40095224	-5.45302571	-5.39009342	-5.38958984
97	-3.54080603	-3.59227005	-3.53026532	-3.52978029
99	-3.54080603	-3.59226883	-3.53026505	-3.52977957
101	-1.43134111	-1.48222524	-1.42136921	-1.42089752
103	-1.43134111	-1.48222363	-1.42136891	-1.42089641
105	-1.17572159	-1.22628397	-1.16604657	-1.16558328
107	-1.17572159	-1.22627266	-1.16604647	-1.16557643
109	-1.17572159	-1.22627081	-1.16604616	-1.16557602
111	-0.98522833	-1.03292673	-0.97786391	-0.97740888
113	-0.53127218	-0.57611273	-0.52526963	-0.52482878
115	-0.19681621	-0.23992686	-0.19028724	-0.18996845
117	-0.19681621	-0.23991441	-0.19028440	-0.18996091
lumo	-0.04877619	-0.16891976	-0.05044183	-0.05066086

References

(S1) PyBertha git URL: `https://github.com/BERTHA-4c-DKS/pybertha` written by: L. Storchi, M. De Santis, L. Belpassi (Accessed: 2022-04-07).

(S2) Jacob, C. R.; Beyhan, S. M.; Bulo, R. E.; Gomes, A. S. P.; Götz, A. W.; Kiewisch, K.; Sikkema, J.; Visscher, L. PyADF - A scripting framework for multiscale quantum chemistry. *J. Comput. Chem.* **2011**, *32*, 2328–2338.

(S3) Jacob, C. R.; Beyhan, S. M.; Bulo, R. E.; Gomes, A. S. P.; Goetz, A.; Handzlik, M.; Kiewisch, K.; Klammler, M.; Sikkema, J.; Visscher, L. PyADF— A Scripting Framework for Multiscale Quantum Chemistry: Version 0.96. 2020; URL: https://github.com/chjacob-tubs/pyadf-releases (Accessed: 2022-04-07), DOI: 10.5281/zenodo.3834283.

(S4) Gomes, A. S. P.; Jacob, C. R. PyEmbed — A Frozen-Density Embedding Module for PyADF. 2020; available at DOI: 10.5281/zenodo.3834283.

(S5) SchmittâĂŘMonreal, D.; Jacob, C. R. FrozenâĂŘdensity embeddingâĂŘbased manyâĂŘbody expansions. *Int. J. Quantum Chem.* **2020**, *120*, e26228.

(S6) Ekström, U.; Visscher, L.; Bast, R.; Thorvaldsen, A. J.; Ruud, K. Arbitrary-Order Density Functional Response Theory from Automatic Differentiation. *J. Chem. Theory Comput.* **2010**, *6*, 1971–1980.

(S7) Ekström, U. XCFun: Exchange-Correlation functionals with arbitrary order derivatives. https://github.com/dftlibs/xcfun (Accessed: 2022-04-07), 2019.

(S8) Baerends, E. J.; Ziegler, T.; Atkins, A. J.; Autschbach, J.; Bashford, D.; Baseggio, O.; Bérces, A.; Bickelhaupt, F. M.; Bo, C.; Boerritger, P. M.; Cavallo, L.; Daul, C.; Chong, D. P.; Chulhai, D. V.; Deng, L.; Dickson, R. M.; Dieterich, J. M.; Ellis, D. E.; van Faassen, M.; Ghysels, A.; Giammona, A.; van Gisbergen, S. J. A.; Goez, A.; Götz, A. W.; Gusarov, S.; Harris, F. E.; van den Hoek, P.; Hu, Z.; Jacob, C. R.; Jacobsen, H.; Jensen, L.; Joubert, L.; Kaminski, J. W.; van Kessel, G.; König, C.; Kootstra, F.; Kovalenko, A.; Krykunov, M.; van Lenthe, E.; McCormack, D. A.; Michalak, A.; Mitoraj, M.; Morton, S. M.; Neugebauer, J.; Nicu, V. P.; Noodleman, L.; Osinga, V. P.; Patchkovskii, S.; Pavanello, M.; Peeples, C. A.; Philipsen, P. H. T.; Post, D.; Pye, C. C.; Ramanantoanina, H.; Ramos, P.; Ravenek, W.; Rodríguez, J. I.; Ros, P.; Rüger, R.; Schipper, P. R. T.; Schlüns, D.; van Schoot, H.; Schreckenbach, G.; Seldenthuis, J. S.; Seth, M.; Snijders, J. G.; Solà, M.; M., S.; Swart, M.; Swerhone, D.; te Velde, G.; Tognetti, V.; Vernooijs, P.; Versluis, L.; Visscher, L.; Visser, O.; Wang, F.; Wesolowski, T. A.; van Wezenbeek, E. M.; Wiesenekker, G.; Wolff, S. K.; Woo, T. K.; Yakovlev, A. L. ADF2019, SCM, Theoretical Chemistry, Vrije Universiteit, Amsterdam, The Netherlands, https://www.scm.com (Accessed: 2022-04-07).

(S9) Dułak, M.; KamiÅĎski, J. W.; Wesołowski, T. A. Linearized orbital-free embedding potential in self-consistent calculations. *Int. J. Quantum Chem.* **2009**, *109*, 1886–1897.

(S10) Dunning, T. H. Gaussian basis sets for use in correlated molecular calculations. I. The atoms boron through neon and hydrogen. *J. Chem. Phys.* **1989**, *90*, 1007–1023.

(S11) Kendall, R. A.; Dunning, T. H.; Harrison, R. J. Electron affinities of the firstâĂŘrow atoms revisited. Systematic basis sets and wave functions. *J. Chem. Phys.* **1992**, *96*, 6796–6806.

(S12) Dyall, K. G.; Fægri, K. Kinetic balance and variational bounds failure in the solution of the Dirac equation in a finite Gaussian basis set. *Chem. Phys. Lett.* **1990**, *174*, 25–32.

(S13) te Velde, G.; Bickelhaupt, F. M.; Baerends, E. J.; Fonseca Guerra, C.; van Gisbergen, S. J. A.; Snijders, J. G.; Ziegler, T. Chemistry with ADF. *J. Comput. Chem.* **2001**, *22*, 931–967.

(S14) Perdew, J. P.; Burke, K.; Ernzerhof, M. Perdew, Burke, and Ernzerhof Reply:. *Phys. Rev. Lett.* **1998**, *80*, 891–891.

(S15) Becke, A. D. Density-functional exchange-energy approximation with correct asymptotic behavior. *Phys. Rev. A* **1988**, *38*, 3098.

(S16) Lee, C.; Yang, W.; Parr, R. G. Development of the Colle-Salvetti correlation-energy formula into a functional of the electron density. *Phys. Rev. B* **1988**, *37*, 785.

(S17) Vosko, S. H.; Wilk, L.; Nusair, M. Accurate spin-dependent electron liquid correlation energies for local spin density calculations: a critical analysis. *Can. J. Phys.* **1980**, *58*, 1200–1211.

(S18) Slater, J. C. A simplification of the Hartree-Fock method. *Phys. Rev.* **1951**, *81*, 385.

(S19) Calaminici, P.; Janetzko, F.; Köster, A. M.; Mejia-Olvera, R.; Zuniga-Gutierrez, B. Density functional theory optimized basis sets for gradient corrected functionals: 3d transition metal systems. *J. Chem. Phys.* **2007**, *126*, 044108.

(S20) Storchi, L. PyBERTHA utilities. https://github.com/BERTHA-4c-DKS/pybertha/tree/master/utils, 2022.

(S21) Virtanen, P.; Gommers, R.; Oliphant, T. E.; Haberland, M.; Reddy, T.; Cournapeau, D.; Burovski, E.; Peterson, P.; Weckesser, W.; Bright, J.; van der Walt, S. J.; Brett, M.; Wilson, J.; Millman, K. J.; Mayorov, N.; Nelson, A. R. J.; Jones, E.; Kern, R.; Larson, E.; Carey, C. J.; Polat, İ.; Feng, Y.; Moore, E. W.; VanderPlas, J.; Laxalde, D.; Perktold, J.; Cimrman, R.; Henriksen, I.; Quintero, E. A.; Harris, C. R.; Archibald, A. M.; Ribeiro, A. H.; Pedregosa, F.; van Mulbregt, P.; SciPy 1.0 Contributors, SciPy 1.0: Fundamental Algorithms for Scientific Computing in Python. *Nature Methods* **2020**, *17*, 261–272.

(S22) Beckstein, O. GridDataFormats package. https://pypi.org/project/GridDataFormats/, 2022.

(S23) Dyall, K. G. Relativistic double-zeta, triple-zeta, and quadruple-zeta basis sets for the 5d elements Hf-Hg. *Theor. Chem. Acc.* **2004**, *112*, 403–409.

(S24) Dyall, K. G.; Gomes, A. S. P. Revised relativistic basis sets for the 5d elements Hf–Hg. *Theor. Chem. Acc.* **2010**, *125*, 97.

(S25) Dyall, K. G. Relativistic quadruple-zeta and revised triple-zeta and double-zeta basis sets for the 4p, 5p, and 6p elements. *Theor. Chem. Acc.* **2006**, *115*, 441–447.

(S26) Dyall, K. G. Relativistic Double-Zeta, Triple-Zeta, and Quadruple-Zeta Basis Sets for the 4s, 5s, 6s, and 7s Elements. *J. Phys. Chem. A* **2009**, *113*, 12638–12644.

(S27) Rampino, S.; Storchi, L.; Belpassi, L. GoldâĂŞsuperheavy-element interaction in diatomics and cluster adducts: A combined four-component Dirac-Kohn-Sham/charge-displacement study. *J. Chem. Phys.* **2015**, *143*, 024307.
